# Assessing Lagrangian inverse modelling of urban anthropogenic CO_2_ fluxes using in situ aircraft and ground-based measurements in the Tokyo area

**DOI:** 10.1186/s13021-019-0118-8

**Published:** 2019-05-17

**Authors:** Ignacio Pisso, Prabir Patra, Masayuki Takigawa, Toshinobu Machida, Hidekazu Matsueda, Yousuke Sawa

**Affiliations:** 1 JAMSTEC, Yokohama, 236 0001 Japan; 2 National Institute for Environmental Studies, Tsukuba, 305 8506 Japan; 3 Meteorological Research Institute, Tsukuba, 305 0052 Japan; 4 Present Address: NILU, 2027 Kjeller, Norway

## Abstract

**Background:**

In order to use in situ measurements to constrain urban anthropogenic emissions of carbon dioxide (CO_2_), we use a Lagrangian methodology based on diffusive backward trajectory tracer reconstructions and Bayesian inversion. The observations of atmospheric CO_2_ were collected within the Tokyo Bay Area during the Comprehensive Observation Network for TRace gases by AIrLiner (CONTRAIL) flights, from the Tsukuba tall tower of the Meteorological Research Institute (MRI) of the Japan Meteorological Agency and at two surface sites (Dodaira and Kisai) from the World Data Center for Greenhouse Gases (WDCGG).

**Results:**

We produce gridded estimates of the CO_2_ emissions and calculate the averages for different areas within the Kanto plain where Tokyo is located. Using these inversions as reference we investigate the impact of perturbing different elements in the inversion system. We modified the observations amount and location (surface only sparse vs. including aircraft CO_2_ observations), the background representation, the wind data used to drive the transport model, the prior emissions magnitude and time resolution and error parameters of the inverse model.

**Conclusions:**

Optimized fluxes were consistent with other estimates for the unperturbed simulations. Inclusion of CONTRAIL measurements resulted in significant differences in the magnitude of the retrieved fluxes, 13% on average for the whole domain and of up to 21% for the spatiotemporal cells with the highest fluxes. Changes in the background yielded differences in the retrieved fluxes of up to 50% and more. Simulated biases in the modelled transport cause differences in the retrieved fluxes of up to 30% similar to those obtained using different meteorological winds to advect the Lagrangian trajectories. Perturbations to the prior inventory can impact the fluxes by ~ 10% or more depending on the assumptions on the error covariances. All of these factors can cause significant differences in the estimated flux, and highlight the challenges in estimating regional CO_2_ fluxes from atmospheric observations.

**Electronic supplementary material:**

The online version of this article (10.1186/s13021-019-0118-8) contains supplementary material, which is available to authorized users.

## Background

Anthropogenic emissions of CO_2_ and the other greenhouse gases (GHGs) impact the atmospheric radiative budget and hence climate [1]. Urbanisation has concentrated more than 50% of the global population, at least 70% of fossil-fuel carbon dioxide emissions (of which nearly 44% direct emission) into a small fraction of the Earth’s land surface [2]. Estimations of CO_2_ fluxes at sub-continental scales contain significant uncertainties (up to 50%), and these uncertainties are larger for finer spatial and temporal scales [3] such as those required for the flux assessment of a single city. Such uncertainty limits the effectiveness of comprehensive mitigation policies at global, regional and national levels. In the so-called “bottom-up” approach, CO_2_ emissions from fossil fuel consumption are estimated based on socio-economic databases [4]. Their accuracy depends on the reliability of information about the consumption of fossil fuels and industrial activities within the studied areas. Therefore, complementary independent assessment is desirable. International agreements to limit greenhouse gas emissions require verification to ensure that they are effective and fair [5]. A concerted effort is needed to transform emerging scientific methods and technologies into an operational monitoring system to support urban carbon management decisions [6].

In situ measurements of atmospheric CO_2_ concentration contain information about upwind CO_2_ surface sources. For a study in Indianapolis long horizontal transects were flown perpendicular to the wind downwind of the city [7]. Emissions were calculated using the wind speed and the difference between the concentration in the plume and the background concentration. The urban plume was clearly distinguishable in the downwind concentrations for most flights. Additionally, there was large variability in the measured day-to-day emissions fluxes as well as in the relative CH4 and CO_2_ fluxes. Turnbull et al. [8] collected in situ measurements and flask samples in the boundary layer and free troposphere over Sacramento, California, USA. The resulting emissions were uncertain to within a factor of two due to uncertainties in wind speed and boundary layer height. Newman et al. [9] used in situ GHG, and planetary boundary layer height measurements recorded in Pasadena, California, USA, to deduce the diurnally varying anthropogenic component of observed CO_2_ in the megacity of Los Angeles (LA). Turnbull et al. [10] used tower flask samples to examine how the choice of background and downwind sampling location can influence estimates of total CO_2_, CO_2_ from fossil fuels, and CO in the urban region of Indianapolis, USA. With background measurements directly upwind of the urban area the local urban emissions could be isolated from other sources. The choice of downwind location and sampling height is also important.

Measurements of atmospheric CO_2_ concentrations and transport model simulations can be used for constraining the surface fluxes by the so-called top-down approach or inverse modelling. In the inverse approach, the atmospheric transport model can be linearised and the transport operator is inverted in order to relate emissions (e.g. anthropogenic) with a measured concentration. Regional (area ∼ 10^4^ km^2^) assessments of fluxes using global models are hindered at small time and space scales due to transport models inability to represent CO_2_ measurements adjacent to large point sources [11]. Therefore, a higher resolution methodology is desirable [6], with grid cells in the range of ∼ 1 km^2^ [12]. Lagrangian-based techniques are well suited for this application, and recent studies are increasingly addressing city-scale inversion problems. Nehrkorn et al. [13] examined the utility of atmospheric observations and models for detecting trends in concentrated emissions from Salt Lake City, Utah, USA. They assessed the ability of different configurations (land surface, planetary boundary layer, and subgrid convective transport) of the Stochastic Time-Inverted Lagrangian Transport model (STILT) [14] to reproduce the observed local and mesoscale circulations and the diurnal evolution of the planetary boundary layer (PBL). They showed that for urban locations there is a clear benefit from parameterising the urban canopy for simulation of the PBL and near-surface conditions, particularly for temperature evolution at night. McKain et al. [15] tested a method for estimating scaling factors with observations from a network of CO_2_ surface monitors in Salt Lake City. They demonstrate an observation-model framework capable of detecting a change in anthropogenic CO_2_ emissions of 15% or more from an urban region on a monthly basis. McKain et al. [15] also argue that integrated column measurements of the urban dome of CO_2_ from the ground and/or space are less sensitive than surface point measurements to the redistribution of emitted CO_2_ by small-scale processes and thus may allow for more precise trend detection of emissions from urban regions. Bréon et al. [16] estimate the Paris area emissions from measurements of atmospheric CO_2_ mol fractions and prior flux inventories. Their analysis is based on measurements from the autumn period because of the reduced interference with biogenic fluxes. More recent studies include Sargent et al. [17] and Babenhauserheide et al. [18].

In this study we estimate CO_2_ flux constraints based on Lagrangian backward transport modeling and a Bayesian inverse method. We present a case study of the Tokyo metropolis, the world’s largest megacity with nearly 40 million inhabitants (for the whole megalopolis in the Kanto plain). Tokyo’s large territorial extent, high population density and intense economic activity create a strong anthropogenic CO_2_ signal. In addition, the fluxes were calculated for the winter months (December to March) when the biospheric activity within the area can be considered dormant and have a smaller impact on CO_2_ mixing ratios than anthropogenic activity [19]. The transport is modeled using ensembles of diffusive backward trajectories [20] using Lagrangian particle dispersion models (FLEXPART, Stohl et al. [21]; flexpart-wrf, de Foy et al. [22], Brioude et al. [23]; TRACZILLA, Legras et al. [24], Pisso and Legras [25]). In order to assess the methodology we repeated our calculations changing a number of input parameters. We used different configurations of the observational constraint, different estimates for the background concentrations, different transport operators (including different input wind fields and perturbations thereof) and different prior emissions derived from both the EDGAR and CDIAC inventories.

## Results

We assessed CO_2_ anthropogenic fluxes from the Tokyo Bay area with observation-based constraints. The top-down estimates are based on a composite data set of CO_2_ observations and a Bayesian inversion methodology. The measurements correspond to two ground sites (Dodaira and Kisai), a tall tower (Tsukuba) and a commercial flight measurement project (CONTRAIL). Night time observations are not used except in sensitivity estimates in order to prevent model biases. The transport operator (source-receptor relationship, or SRR) is calculated using backward Lagrangian calculations based on ECMWF (European Center for Medium-Range Weather Forecasts) winds. The background CO_2_ can be obtained from the measurements themselves (although alternative representations have been tested, see “Methods” section). The prior anthropogenic fluxes are based on the EDGAR and CDIAC CO_2_ inventories. We fist present examples of the reference inversions and an analysis of the multi year set of measurements. A series of sensitivity tests have been carried out using different subsets of the measurements, perturbations to the transport operator and different background representations.

### Emission flux inversion and simulated mixing ratio calculation

**Fig. 1:**
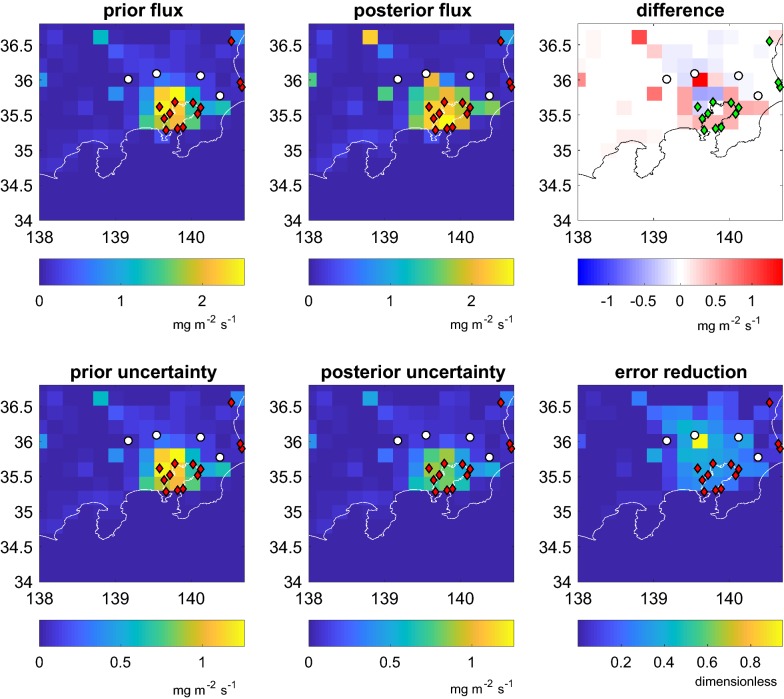
Prior and posterior fluxes averaged for the whole period with the corresponding averaged flux uncertainties. The upper row shows the monthly mean per each grid cell for prior flux (left), posterior flux (center) and its difference (right). The lower row shows the monthly mean per each grid cell for prior uncertainty (left), posterior uncertainty (center) and the error reduction (right). The error reduction is calculated daily and averaged monthly. All averages were calculated from daily retrievals for the period 2005–2009

Figure 1 shows the result of the averaged inversions for all winters 2005–2009. The upper row left and centre panels show respectively the prior and posterior fluxes. The constraints were calculated omitting night time observations from all platforms, observation-based background, ECMWF winds, the EDGAR anthropogenic prior fluxes and the error covariance matrices are described in “Methods: description of the data and numerical models” section. We calculated the posterior fluxes and posterior flux uncertainties assuming Gaussian errors [26]. The upper right panel shows the space distribution of the difference between posterior and prior fluxes. The lower row presents the prior and posterior flux uncertainties estimated as the square root of the error variances (i.e. the square root of the diagonal of the error covariance matrices **B** and **B**_**0**_, see “Methods” section). The upper right panel shows the error reduction, a metric for the difference between prior and posterior uncertainty discussed in “Prior flux error covariance matrix” section.

**Fig. 2:**
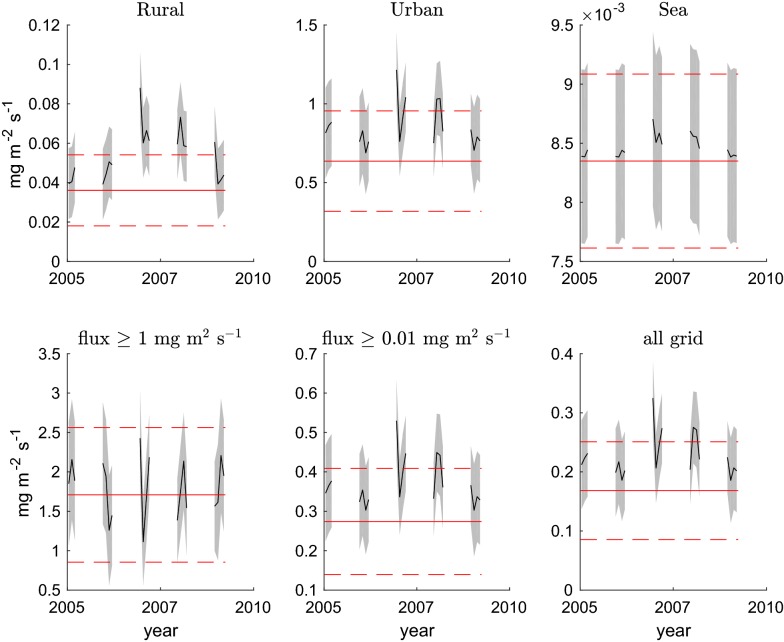
Time series of spatially averaged fluxes for 2005–2009. Upper row: rural, urban and sea domains. Lower row: areas corresponding to EDGAR grid cells with flux higher than 1 mg CO_2_ m^2^ s^−1^ (left panel) and 0.01 mg CO_2_ m^2^ s^−1^ (center panel) and inner domain. The masks are those shown in Additional file 1: Figure S1. The black lines represent the posterior fluxes. The grey shaded area represents 1-sigma for the posterior uncertainty. The red solid and dashed lines correspond to the mean flux and 1-sigma uncertainty for the prior

Based on the daily averaged maps of optimised fluxes, we calculated various spatial averages of the prior and posterior fluxes. Figure 2 illustrates the space averaged flux values using different domains and grid masks. The masks used for the spatial averages are those shown in Additional file 1: Figure S1. Rural areas are defined for the purposes of these calculations as the land grid cells where typically the EDGAR anthropogenic fluxes are lower than the Vegetation Integrative SImulator for Trace gases (VISIT, [27]) biogenic fluxes. Urban areas are the complement of the rural areas over the land. Sea and land masks are defined to be consistent with WRF output at a 10 km horizontal resolution. We have included the spatial averages taken over three additional masks for comparison. The lower left panel shows the averages taken daily on the grid cells where the EDGAR flux is higher than 1 mg CO_2_ m^−2^ s^−1^. The lower central panel where the EDGAR fluxes are higher than 0.01 mg CO_2_ m^−2^ s^−1^. The lower right panel shows the average over the whole grid in the inner nest centred in Tokyo used for the inversion (138° E to 141° E and 34° N to 37° N). In general the posterior averages are larger than the priors.

The averaged density and the total flux integrated in the regions defined above (and Additional file 1: Figure S1) for the whole period 2005–2009 are shown in Tables 1 and 2.

**Table 1:** Total flux time and space averages for the whole period 2005–2009

	Prior flux	Posterior flux	Prior uncert	Posterior uncert
Rural	34	50	17	16
Urban	411	554	205	161
Sea	9	9	< 1	< 1
≥ 1 mg m^−2^ s^−1^	276	291	138	105
≥ 0.01 mg m^−2^ s^−1^	453	612	222	178
All grid	455	612	224	179

All values are in Mt CO_2_ y^−1^ integrated in the area defined by the mask

**Table 2:** Flux density time and space averages for the whole period 2005–2009

	Prior flux	Posterior flux	Prior uncert	Posterior uncert
Rural	0.82	1.22	0.41	0.41
Urban	14.47	19.46	7.23	5.67
Sea	0.19	0.19	0.02	0.02
≥ 1 mg m^−2^ s^−1^	38.84	40.93	19.42	14.80
≥ 0.01 mg m^−2^ s^−1^	6.23	8.41	3.06	2.44
All grid	3.82	5.16	1.88	1.50

All values are in micro-mol CO_2_ m^−2^ s^−1^ commonly adopted for CO_2_ fluxes in the eddy covariance community (1 micro-mol CO_2_ = 0.044 mg CO_2_, the corresponding Additional file 6: Table S1 in mg CO_2_ m^−2^ s^−1^ can be found). Space averages are taken in the area defined by the mask

Moriwaki and Kanda [28] obtained averaged flux values in winter of 0.25 mg CO_2_ m^−2^ s^−1^ (range between 0.2 and 1.1 mg CO_2_ m^−2^ s^−1^) based on direct micrometeorological measurements made from May 2001 to April 2002 in a low-storied residential area in Kugahara, Tokyo, Japan (35.5667 N, 139.6833 E). These measured flux values provide a range of a priori fluxes in mixed urban areas in Tokyo during the period under consideration. The flux values obtained here interpolated in the area where these experiments took place are consistent with this estimate, although the comparisons being made between point-wise measurements and a large area inversion. Our estimates are strongly affected by the a priori baseline and other factors as further explained below.

**Fig. 3:**
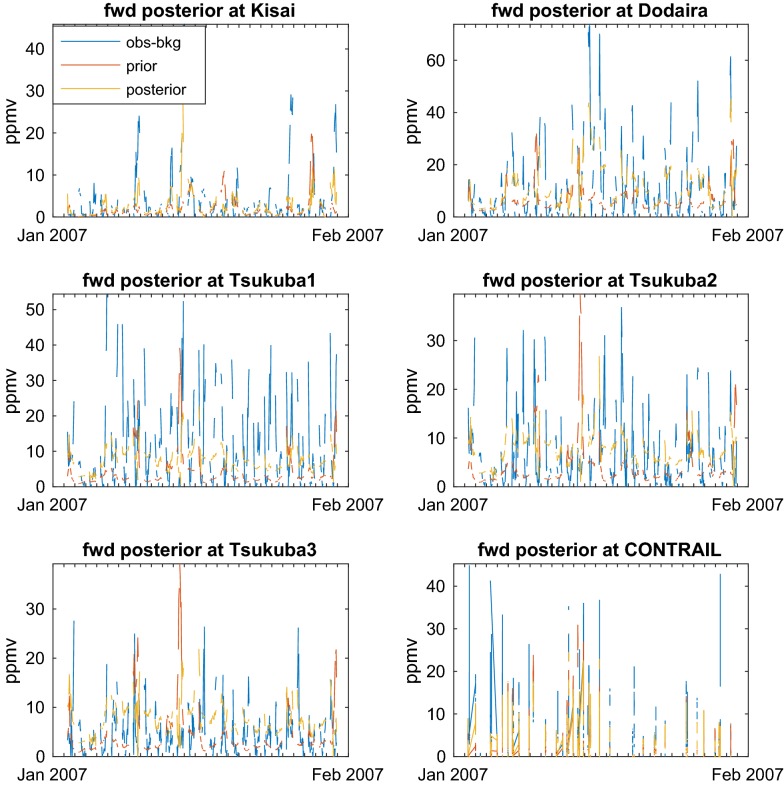
Comparison of the CO_2_ values for the measurements and the forward model based on prior and posterior fluxes for a reference monthly inversion (January 2007). The time series correspond Kisai (13 m.a.s.l.) and Mt. Dodaira (840 m.a.s.l.), the three levels of the Tsukuba tower (base at 33 m.a.s.l., inlets at 25, 100, and 200 m above ground level) and the composite of the CONTRAIL data (variable heights from ~ 500 to 2000 m.a.s.l)

Figure 3 shows the observed CO_2_ values compared with the prior and posterior forward models for January 2007. The measurements for January 2007 were separated into six time series corresponding to the stations at Kisai (13 m.a.s.l.) and Mt. Dodaira (840 m.a.s.l.), the three levels of the Tsukuba tower (base at 33 m.a.s.l., inlets at 25, 100, and 200 m above ground level) and the composite of the CONTRAIL data (variable heights from ~ 500 to 2000 m.a.s.l).

Hourly averaged data is used for Kisai, Dodaira and Tsukuba. We avoid using the nighttime data due to lack of confidence in nocturnal simulations. CONTRAIL measurements are carried out continuously. The bottom altitudes of the ascents and descents, near the Narita runways, are removed from the analyses to prevent highly localized contamination. Observations from layer of high values above the Narita airport was also removed. CONTRAIL commercial flights take place daily (see Additional file 2: Figure S2 and Additional file 3: Figure S3 for the vertical distribution and the hourly distribution within the day). The CO_2_ values for the measurements are compared with the source–receptor relationship used as forward model applied to both prior and posterior fluxes. It can be seen from Fig. 3 that the model performs consistently better with the posterior fluxes than with the prior in every time series. For a more quantitative assessment we have calculated the correlation coefficients together with their significance p-values for the individual time series and for the full data composite. The results are displayed in Table 3. All p values indicate an acceptable level of significance for the correlations displayed in the table. The inversion calculation improves the correlations for all measurement time series individually. Taken as a whole, the correlation coefficient improves from 0.18 to 0.6. However, the model does not always capture the highest peaks. In these extreme cases, the errors in the modelled mole fractions can be of the order of magnitude of the signals.

**Table 3:** Observed CO_2_ concentrations compared with prior and posterior model results: correlation coefficients and corresponding p-values for the reference inversion in January 2007

	Corrcoef prior	p (significance) prior	Corrcoef posterior	p (significance) posterior
Kisai	0.09	0.14	0.48	< 0.01
Dodaira	0.02	0.65	0.73	< 0.01
Tsukuba 1	0.02	0.61	0.54	< 0.01
Tsukuba 2	− 0.06	0.16	0.41	< 0.01
Tsukuba 3	− 0.07	0.10	0.29	< 0.01
CONTRAIL	0.34	< 0.01	0.58	< 0.01
All data	0.18	< 0.01	0.60	< 0.01

### Sensitivity to different inversion system parameters

The inversion results depend on the parameters for the different components of the system. These parameters include the choice of the subset of measurements, the background concentrations assumed in the individual observations time and locations, the random errors and biases in the transport models, and the a priori fluxes. The month of January 2007 was chosen because it is the one for which all tested options are available: aircraft, tower and ground observations, AGCM simulations for the background and WRF simulations for the transport. Not every flux inversion from the perturbations provides a necessarily realistic estimate of the emissions, but the results in this section should be interpreted as sensitivity experiments for future model calibration and comparison.

#### Sensitivity to measurement amount and location

**Fig. 4:**
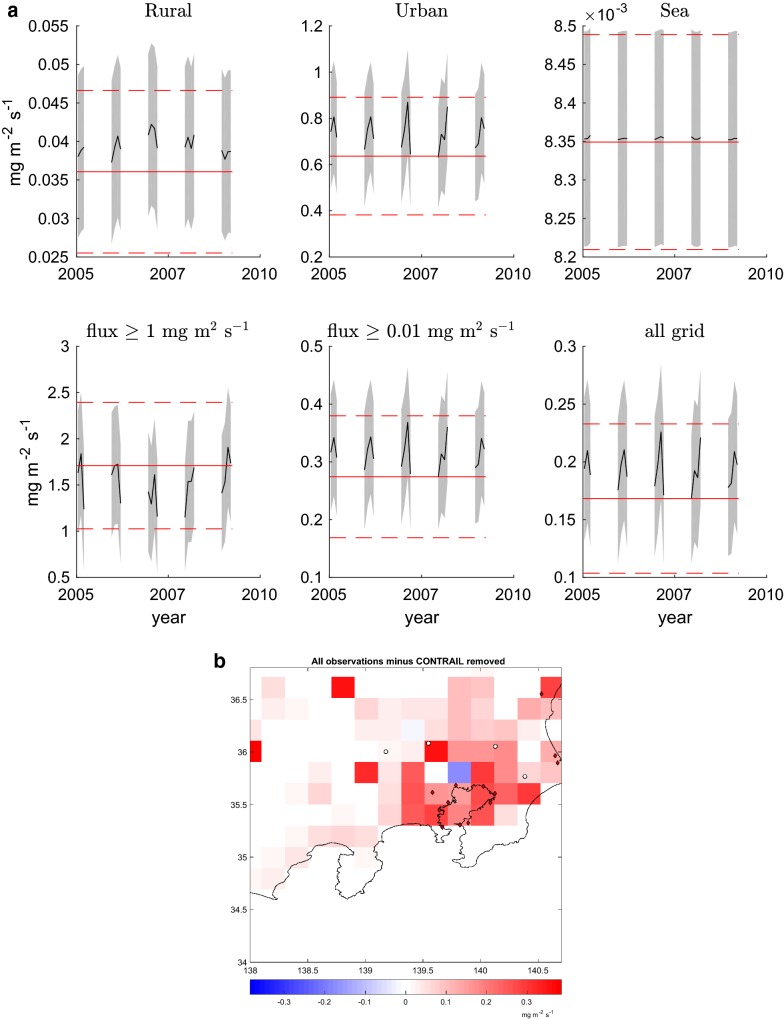
Impact of CONTRAIL. **a** Time series of averaged fluxes with the masks described in the Additional file 1: Figure S1 of the retrieval obtained omitting the CONTRAIL data. **b** Spatial distribution of the difference between the retrievals obtained with all the observation and the retrievals obtained with CONTRAIL removed averaged for the whole period 2005–2009

The use of different subsets of the data yields different flux estimates. We studied the impact of CONTRAIL data on the geometry of retrieved fluxes. Figure 4a shows the difference between the retrievals using all data including CONTRAIL with respect to the results based on ground observations only. In the most urbanised region of Tokyo the difference is up to 21% higher using all data measurements including CONTRAIL. The difference is larger in the central areas where the fluxes are larger. Table 4 shows the impact on the total integrated emissions in the urban area with respect to the reference inversion. For the urban area grid is 13%. This illustrates to what extent the availability of data has a large impact on flux inversion results. Figure 4b shows the evolution in time of fluxes calculated without the CONTRAIL data with night time removed. The largest difference appears in the middle of the interval studied.

**Table 4:** Perturbation tests for January 2007

Experiment description	Kanto urban emissions Mt/month (all period average)	Relative difference with respect to reference
Prior (EDGAR)	34	− 26%
Reference inversion	46	0%
Changes in observations amount and location		
No CONTRAIL	40	− 13%
Including night observations	41	− 11%
Perturbations in transport modelling		
Space shift 1 grid cell [0 1]	56	21%
Space shift 2 grid cells [0 2]	61	32%
Time shift 1 h [1 0]	52	13%
Time shift 2 h [2 0]	54	17%
WRF meteorology	57	23%
Different background representations		
Mauna Loa (ML)	71	54%
ML + 2 ppm	58	26%
ML − 2 ppm	84	82%
AGCM-BDE (i.e. with backward diffusive ensembles)	53	15%
AGCM-BDE + 2 ppm	40	− 13%
AGCM-BDE − 2 ppm	67	45%
Inventory		
2× EDGAR	49	7%
3× EDGAR	50	9%
CDIAC	33 (from prior 11)	− 28% (+ 300% from prior)
EDGAR + VISIT	47	2%
EDGAR + CASA	47	2%

The changes are in the observations amount and location, perturbations in transport modelling, different background representations and inventory perturbations. The baseline inversions used as control correspond to all observations night time excluded, ECMWF winds with no SRR shift, observations-derived background and EDGAR inventory

#### Sensitivity to background concentration representation

**Fig. 5:**
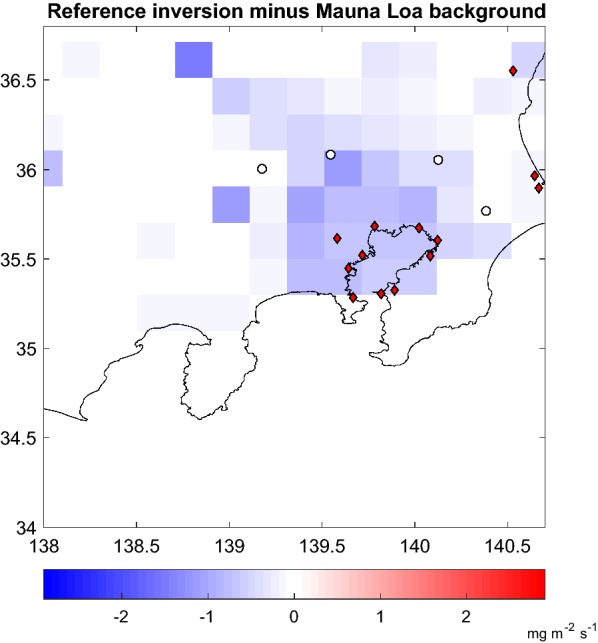
Impact of the background on the retrievals: difference of the average flux for the whole period 2005–2009 between the reference inversion and the inversion using the Mauna Loa interpolated data as background

The regional inversion system needs initial and boundary conditions. The background CO_2_ concentration can be defined as the fraction already present in the atmosphere before the emissions take place. It is a defining parameter in any inversion methodology as it determines the increase ΔCO_2_ that is the input of the inversion operator. Different background estimates yield different flux constraints. Bias in the background translates into a flux estimate error as different background estimates yield different observational constraints on the fluxes. Several papers have discussed the definition and the impact of erroneous boundary conditions in regional inversions [29, 30]. The estimate of the background for the reference simulations can be obtained directly from the data, by taking the daily minimum for each ground site or using the free troposphere observations of CONTRAIL. We tested in addition two other different approaches: using the simple hemispheric seasonal baseline from a clean air station and a global Eulerian model together with ensembles of backward diffusive trajectories. Using Lagrangian transport, the definition of background mixing ratio values depends on the time and space scales under consideration (i.e. how far back the trajectory ensembles are followed) in the presence of emissions. In the case of Tokyo the basis background is related to the seasonally averaged values in the Northern Hemisphere. For the rather usual westerly wind conditions, influx from continental Asia could be non negligible. But as shown by Tohjima et al. [19, Figs. 3 and 8] from both Lagrangian and Eulerian transport representation, the North-East Asian plume has a relatively little impact on Japan in general and on the Tokyo Bay Area in particular. Figure 5 shows the difference between the reference inversion and the inversion done using the clean air site as background. The difference in the retrieved flux is negative throughout the domain: as the clean air site has lower concentrations, the inversion assigns larger fluxes to the domain. The flux retrievals are listed in Table 4. The perturbed calculations for January 2007 include changing the observation-derived background for that from the clean air site (ML) and from AGCM (interpolated and together with EDBTs). In addition, we have calculated the flux resulting from perturbations (offsets) to the different backgrounds of 2 ppm in either direction. Not in all cases the global model output is better than the clean side observations (e.g. Mauna Loa) for background estimates in regional CO_2_ flux inversion. This depends on the calibration of the background of the global model itself: ML + 2 ppm is closer to the reference than AGCM-BDE − 2 ppm. However, if the bias in the background level can be removed, other sources of uncertainty (such as those arising from transport) could have larger effect on the results than the background bias.

#### Sensitivity to transport model errors and biases

**Fig. 6:**
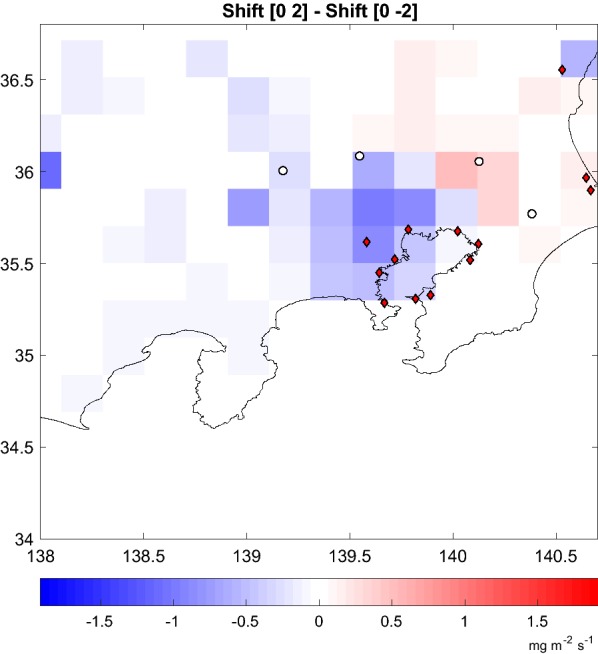
Transport uncertainty: difference of the average flux for the whole period 2005–2009 between the inversion carried out with the SRR shifted two columns to the right (shift [0 2]) minus the inversion carried out with the SRR shifted two columns to the left (shift [0 − 2])

The calculations are sensitive to transport errors that can occur in space and time. We have investigated the impact of biases in the winds on the estimated fluxes. The biases can be represented as changes in the transport operator by perturbing the linear source–receptor relationship. The perturbed runs use a simple shift of the SRR in order to simulate errors in time (columns) and in space (rows). A shifting of the columns of the SRR to the right (left) displaces in the spatial footprint pattern to the east (west) (but causes no change in time if the SRR is calculated for e.g. static fluxes). A shifting the rows downward (upward) causes a delay (advance) in the transport time but little change in the spatial footprint pattern. We retrieved the fluxes with the SRR shifted by 1 and 2 rows and columns keeping otherwise the same parameters of the reference inversion. The resulting retrieved flux differences can be found in Table 4. A space shift of one and two columns (20 and 40 km) causes a difference in the retrieved fluxes of 21% and 32% respectively. A time shift of one and two rows (~ hours) cause a difference of 13% and 17% respectively. Figure 6 shows the difference of the average flux for the whole period 2005–2009 between the inversions carried out with the SRR shifted two columns to the right (shift [0 2]) with respect to shifting two columns to the left (shift [0 − 2]). It is apparent that the flux pattern is displaced to the North East.

In order to compare the effect of different meteorologies, we performed the reference calculation using flexpart-wrf calculated SRRs instead of the default ECMWF. The integrated difference on the residence times in the SRRs ranged between 10 and 15% (“Methods: description of the data and numerical models” section, Additional file 4: Figure S4). Nevertheless the impact on the retrieved flux can be higher, up to 23% for the 1 day in January 2007. The change in meteorology could act in a similar way as the shift: displacing the sensitivity to adjacent cells. If the highest sensitivity is associated with a cell that contains a very low prior flux, the retrieval could need to assign a very large increase to such a cell in order to satisfy the constraint.

#### Sensitivity to the prior flux inventory

The key ingredient regularising the inversions are the prior fluxes. We carried out large perturbations to the a priori EDGAR emission inventories (2× and 3×). When 2×EDGAR and 3×EDGAR were adopted for the a priori emissions, the atmospheric inversion resulted in a difference of 6% and 9% with respect to the reference respectively. In addition we retrieved the flux with the CDIAC inventory. The prior is much lower that that of EDGAR (11 vs. 34 Mt month^−1^). However, the resulting posterior integrated flux en the urban areas is 33 Mt month^−1^ showing that the observations provide and effective constraint for the fluxes. We carried out sensitivity tests in order to estimate the impact of neglecting the biogenic fluxes. We used biogenic priors based on CASA and VISIT (see “Methods: description of the data and numerical models” section). In both cases the difference with respect to the reference inversion was around 2%. In the Tokyo Bay Area during the period of this study, the biogenic fluxes (below 0.1 mg m^−2^ s^−1^) represent just a small fraction of the anthropogenic emissions (up to 3 mg m^−2^ s^−1^). Therefore their impact on the final inversion result during the period of this study is modest with respect to that of the anthropogenic fluxes.

## Discussion

The main limitations of our approach are the treatment of the background and the transport uncertainties. Other limitations include the sparse distribution of measurements and limited availability of meteorological flux measurements for direct flux comparison. However, the simplified settings chosen for this study allow the evaluation of several aspects of the methodology. This yields in turn an elementary characterisation of potential avenues for improvement. The combination of several different transport models with in situ measurements from different inhomogeneous data streams including from commercial aircraft is especially promising.

Transport uncertainties: Meteorological winds are provided by different models (ECMWF and WRF) seem to agree with errors in the SRR of the order of 10–15% in certain experiments. The retrieval process can increase this error in an additional 5–10% akin to a shift perturbation to the SRR. Even if their meteorological winds are provided by different centres (NCEP and ECMWF), the data on which these are based (e.g. satellite radiances for the assimilation processes) are not independent. Hence, there could be biases in the general weather patterns due to the erroneous model representation of weather systems, fronts and other large-scale atmospheric transport structures. On a smaller scale there could be biases introduced by the limited grid cell resolution. The sources of error related to transport include the impact of the PBL parametrisation. The construction of the source–receptor relationship involves the translation of 2-D flux densities to 3-D mixing ratios or concentrations. The SRR can be interpreted as a discrete version of the Green’s function for the transport-diffusion equation. The Green’s function method provides solutions for the transport-diffusion equation with arbitrary initial/boundary conditions as the sum of single impulse-response solutions (i.e. Dirac’s delta functions, that are here analogous to ensembles of Lagrangian trajectories). The discretisation for the Green’s function method is best suited to represent probability transitions between regions of the same dimension (i.e. 3-D to 3-D). Although a rigorous formulation exists for the consideration of 2D boundary fluxes for mixed Neumann–Dirichlet boundary conditions [31, 32], it is not well suited for numerical computations. This is because such a formulation requires the calculation of the Green’s function gradient at the boundary of the domain, which can result in a large error. Instead, an additional step is usually performed for the parameterisation of the mixing within PBL. In this study we have used a mixing height consistent with the 3-D transport model used for advection (ECMWF or WRF). The use of measurements to constrain PBL height is limited by the spatial distribution required within the inversion area. For the present case, suitable measurements to follow this approach are not available. The discrepancy between the model resolution and the real scale of the physical processes may be responsible for representation errors. Given the small scale required for city size flux estimations, inadequate spatial and time resolution can result in errors in the transport model. The compliance with a Courant–Friedrichs–Lewy type condition therefore is required: i.e. the particles must be sampled in an interval shorter than the time for the trajectories to travel to adjacent grid points cells. For example, if wind speed is lower than of 20 km h^−1^, and the horizontal grid is 20 km, then the required time step for the output of trajectories would be 1 h. This is in the range of the transport timescale between the sites of Kisai or Narita and the center of Tokyo. We have performed detailed comparisons between our SRR calculations and the standard FLEXPART output based on a 15-min advection time step and a turbulent perturbation time step of 18 s. The difference found was lower than 5%, which is small considering the other sources of error. We conclude that hourly footprints are sufficient and wouldnot introduce significant biases in this case. Although we attempted to assess the impact of the biases in the transport modeling, there is much space for improvement. Nehrkorn et al. [13] reports that simulation of near-surface CO_2_ concentrations for a 2-week period in October 2006 showed that running WRF at high resolution (1.33 km) and with an urban canopy model improves the simulation of CO_2_. Future runs with improved mesoscale model parametrisation are expected to yield more accurate results. As the aircraft crosses the top of the boundary layer, airborne observations are sensitive to errors in the representation of vertical mixing in the transition. The CONTRAIL data could be converted into vertically integrated atmospheric column amounts (XCO_2_) and adopted within the inversion. Using XCO_2_ could help reduce sensitivity to model errors, and will be explored in future work.

Background mixing ratios are a key element and poorly constrained in the current study. In out case, the use of a background that takes into account mainland Asian continental emissions yields an estimate that is lower than EDGAR inventory in the most urbanised areas (the centre of Tokyo where EDGAR emissions are higher than 1 mg CO_2_ m^−2^ s^−1^). In contrast, the use of a clean air site as background leads to the conclusion that the inventories underestimate the fluxes. In agreement with Turnbull et al. [10], in this case it is most likely that the measured increase in CO_2_ in not only originated from TBA emissions but that the enhancement in CO_2_ is from both TBA emissions with some from surrounding areas. Previous studies have signalled the uncertainties associated with the background. For Indianapolis in winter, total CO_2_ enhancements relative to the background from the surrounding rural land are almost entirely due to fossil fuel CO_2_ (CO_2_ff) so that CO_2_ enhancement can be used as a proxy for CO_2_ff. In contrast, when a free tropospheric or continental clean air background site is used, CO_2_ff contributes only about half of the CO_2_ enhancement downwind of Indianapolis [10], see also Lauvaux et al. [33]. Thus, raw CO_2_ enhancement will frequently not be a good proxy for CO_2_ff when a continental background is used. Bréon et al. [16] reports that the boundary concentration for Paris is underestimated when wind comes from North west (The Benelux). In Los Angeles, local fossil fuel combustion contributed up to 50% of the observed CO_2_ enhancement overnight, and 100% of the enhancement near midday [9].

We have found that even using used in addition of aircraft data a combination of ground in situ measurements and tower data the amount and distribution of input measurement data has a large impact on the results of the inversions. Mays et al. [7] underline the uncertainty arising from inadequate spatial sampling. Turnbull et al. [10] observes that when measurements are made too far downwind, both plume dispersion and the relatively small proportion of the time that the location samples the plume reduce the detectability of the urban signal. On the other hand, the Salt Lake City case [15] suggests that increasing the number of surface measurement stations across the city would be ineffective at substantially improving the observational approach for detecting a change in emissions. Simulations in that case indicate that individual observation sites are sensitive to emissions across the full urban region. Turner et al. [34] discuss tradeoffs between measurement density and flux accuracy. The airborne measurements provide additional information to assess such a flux variability that may be missed using only ground or tower data. Several studies have used aircraft data. Our analysis add to those of Mays et al. [7] in Indianapolis and Turnbull et al. [8] in Sacramento confirming the utility of aircraft based platforms. Mays et al. [7] found that the downwind concentration values clearly show the urban plume in each case, and that the plume concentrations are well above the uncertainty in the background concentrations. The CONTRAIL flights were borne on commercial airliners, so we lack specific upwind and downwind transects. From the technical point of view, we developed a system that merges aircraft data together with ground in situ measurements and tower data.

The scarcity of the measurements limits the assessment of the spatiotemporal variability. Mays et al. [7] indicate significant variability in the fluxes of CO_2_ from Indianapolis. Comparison with measurements of the forward model (Fig. 3) has been carried out as in the study of Bréon et al. [16]. As in their case, the errors in the modelled mole fractions can be of the order of that of the signals for the largest peaks (see “Results” section).

For heavily vegetated cities, it is necessary to distinguish anthropogenic from biogenic emissions, possibly with tracer measurements of fossil fuel combustion (e.g. CO, 14C) [15]. The region surrounding Indianapolis has a strong seasonal biogenic CO_2_ cycle, with a dormant biosphere in winter and strong biospheric exchange in summer [10]. The analysis of Bréon et al. [16] is based on measurements from the autumn period. It helps the inversion of fossil fuel emissions because of the reduced interference with biogenic fluxes. Ye et al. [35] studied biospheric CO_2_ contributions on urban inversions with Observing System Simulation Experiments and NASA’s Orbiting Carbon Observatory 2 (OCO-2) observations. In the case of Tokyo in winter the impact of vegetation is overwhelmed by large anthropogenic emissions. The comparison may be relevant because as in the case of Paris, Tokyo is densely populated and the emissions are intense over a limited surface. Available direct micrometeorological measurements during the same season [28] are consistent with the values presented here, although the comparison can be made only in limited interpolated locations.

In any Bayesian methodology [36, 37], the choice of the anthropogenic flux inventory influences the posterior estimate. The resolution of the inventories used in this study was chosen to be coarse in order to test the methodology. In the future we will use improved inventories such as FFDAS [38] and ODIAC [39]. As in the study of Bréon et al. [16] the prior estimate of CO_2_ doesn’t account for human respiration. Improved assessments of large city CO_2_ fluxes can benefit from the combination of in situ measurements, inventory optimisation and the use of remote sensing such as satellite column integrated measurements.

**Fig. 7:**
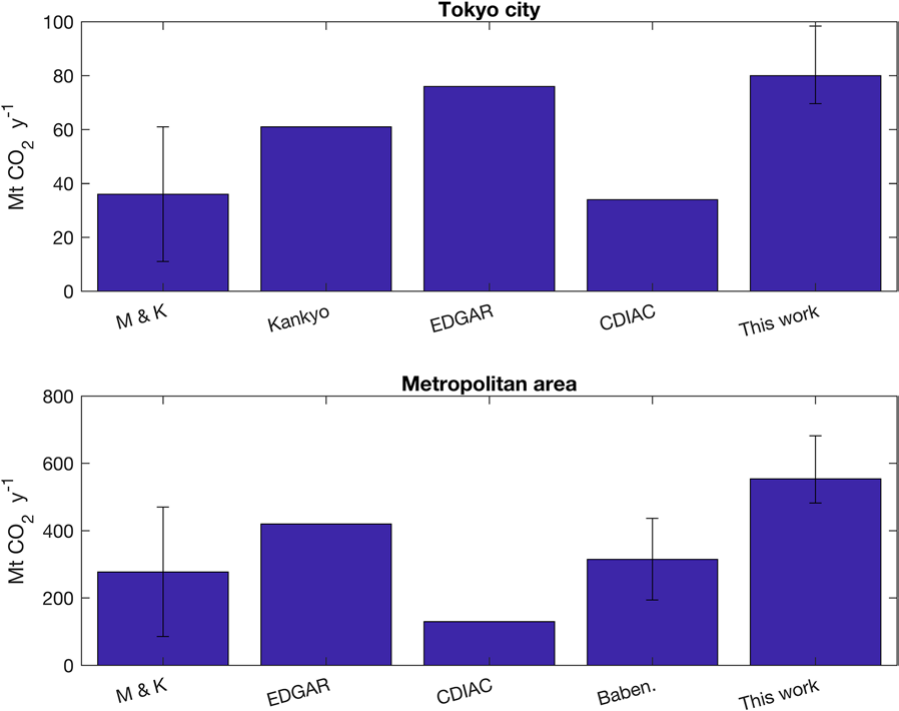
Comparison with literature estimates of Tokyo CO_2_ emissions in units of millions of metric tons of CO_2_ per year (MMT CO_2_ y^−1^ or Mt y^−1^). See also Table 5. M & K: Moriwaki and Kanda [28], Kankyo: Tokyo government, mean 2005–2009, EDGAR and CDIAC for the year 2005, Baben.: Babenhauserheide et al. (in review), This work: uncertainties from “Sensitivity to measurement amount and location” and “Sensitivity to background concentration representation” sections. See also [40]. Hypothetical background perturbations not considered for uncertainty estimates. When inventories are provided without uncertainties, error ranges are not included

Figure 7 shows a comparison with literature estimates of Tokyo CO_2_ emissions in units of millions of metric tons of CO_2_ per year (MMT CO_2_ y^−1^ or Mt y^−1^). The area for Tokyo city is 1808 km^2^ that is the continental Tokyo prefecture (Tokyo-tō). The definition of the Metropolitan area is 13,555 km^2^, that of the city of Tokyo plus the three surrounding prefectures (Ittō-sanken). The fluxes of Moriwaki and Kanda [28] were extrapolated based in their range for winter. The Tokyo government estimates are the average for the years of this study.

**Table 5:** Comparison with literature estimates of Tokyo CO_2_ emissions in units of millions of metric tons of CO_2_ per year (MMT CO_2_ y^−1^ or Mt y^−1^). See also Fig. 7

	Tokyo city	Metropolitan area
Moriwaki and Kanda [28]	11–62 Mt y^−1^	85–470 Mt y^−1^
Tokyo government (Kankyo, mean 2005–2009)	61 Mt y^−1^	N/A
EDGAR	76 Mt y^−1^	420 Mt y^−1^
CDIAC	34 Mt y^−1^	130 Mt y^−1^
Babenhauserheide (in review)	N/A	315 ± 121 Mt y^−1^
This work [40]	80 Mt y^−1^	554 Mt y^−1^

## Conclusions

In this study we assessed an inversion methodology for the anthropogenic CO_2_ emissions of the Tokyo Bay area. In the past, studies had been focused on smaller areas. Recently, larger area cities have been assessed in the context of a coordinated pilot project for the megacities of Los Angeles, Paris and São Paulo. This independent study attempted to address the CO_2_ flux inversion in the urban area of Tokyo assessing the related uncertainties. We applied a Bayesian inversion technique combining Lagrangian particle dispersion model in backward mode with a composite of CO_2_ measurements including ground sites, tall tower and aircraft data. We examined the impact on our results of using different parameters carrying out sensitivity tests. We compared the resulting flux estimates using only sparse surface CO_2_ data vs. including aircraft (CONTRAIL) observations. We used different estimates of the background concentrations (from the data, a clean air station, the ACTM global model and diffusive backward reconstructions with TRACZILLA). We tested different simulated transport biases and used different wind data to drive the transport models (FLEXPART, flexpart-wrf). All of these factors are shown to cause significant differences in the estimated flux. This highlights the challenges in estimating regional CO_2_ fluxes.

Our key results can be summarised as follows:

- The constraints on the spatial distributions obtained using all data including CONTRAIL aircraft data with respect to inversions calculated using ground sites only differ significantly. Differences in inverted fluxes for the whole region amounted to 13% on average and up to 21% in the highest flux cells adding aircraft data from the CONTRAIL dataset.
- Errors in the modelled meteorological transport largely affect the flux estimates. Among the tested case studies, the impact of using different meteorologies (23% on fluxes from 10 to 15% in the residence times) is comparable to shifting in the transport patterns of between 20 and 40 km (21% and 32% respectively).
- Assumed background concentrations impact the results and must be assessed. Background concentrations impacts were determined by the linearised transport operator. Replacing the background obtained directly from the observations for those calculated form a clean air station and a global Eulerian model (AGCM) amounted to differences of 54% and 15 respectively.
- Replacing the EDGAR inventory with CDIAC yielded emissions 28% lower. Using 2×EDGAR and 3×EDGAR yielded emissions 7% and 9% higher. These values were obtained with diagonal terms of the observation error covariance matrix corresponding to 1-sigma values of the order on 1 ppmv and diagonal terms of the prior error covariance matrix corresponding to 1-sigma values of the order of 100%. If the observational constraint is reduced by including the advection error in the diagonal terms of the observation error covariance matrix the retrieved fluxes are much closer to the priors.
- Our estimates of total emissions for the years studied are on average of 80 Mt CO_2_ for the city of Tokyo (continental Tokyo-tō) and 554 Mt y^−1^ for the whole Kanto region.

## Methods: description of the data and numerical models

### Description of the urban area selected for the study

The Tokyo Bay Area (TBA) is located in the Kanto region of Japan, which includes and surrounds the Greater Tokyo Area and encompasses seven prefectures: Gunma, Tochigi, Ibaraki, Saitama, Tokyo, Chiba, and Kanagawa. The region has varied topography and a complex coastline in the East. Within its boundaries, slightly more than 45% of the land area is the Kanto Plain. The rest consists of the hills and mountains that form the land borders. It is a highly developed area with a significant industrial activity and a complex transportation network. The population was about 42 million inhabitants according to an official census count on October 1, 2010 by the Japan Statistics Bureau. This corresponds to approximately one-third of the total population of Japan. The anthropogenic CO_2_ emissions from Tokyo are both large and distributed over an extensive area. The anthropogenic signal is substantially higher than the background and biogenic sources within the most urbanised area, especially during the winter months. The availability and quality of data from the TBA, the largest urban area in the world, makes it suitable for benchmarking inversion methodologies.

### Measurements: CO_2_ mixing ratios measured in commercial aircraft, tower and surface stations

**Fig. 8:**
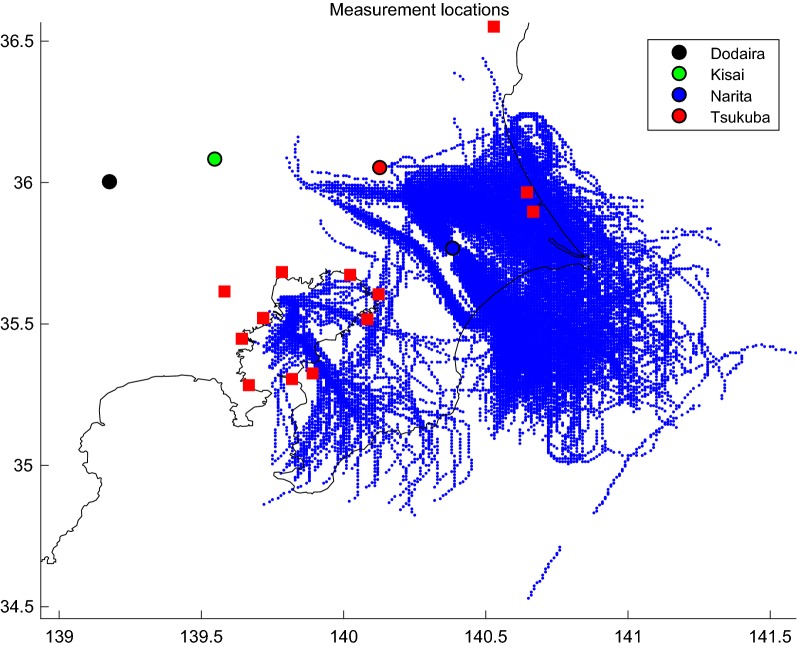
Sampling location for the measurements used in this study and main CO_2_ sources. The blue dots represent the geographical distribution of the CONTRAIL data. The location of the stations of Kisai (13 m.a.s.l.) and Mt. Dodaira (840 m.a.s.l.), the Narita airport (43 m.a.s.l.) base for CONTRAIL flights (observations from ~ 500 m.a.s.l to 2000 m.a.s.l and the Tsukuba tower (base at 33 m.a.s.l., inlets at 25, 100, and 200 m above ground level) are represented by the black, green, blue and red dots respectively. The red squares represent the location of the major power plants

**Fig. 9:**
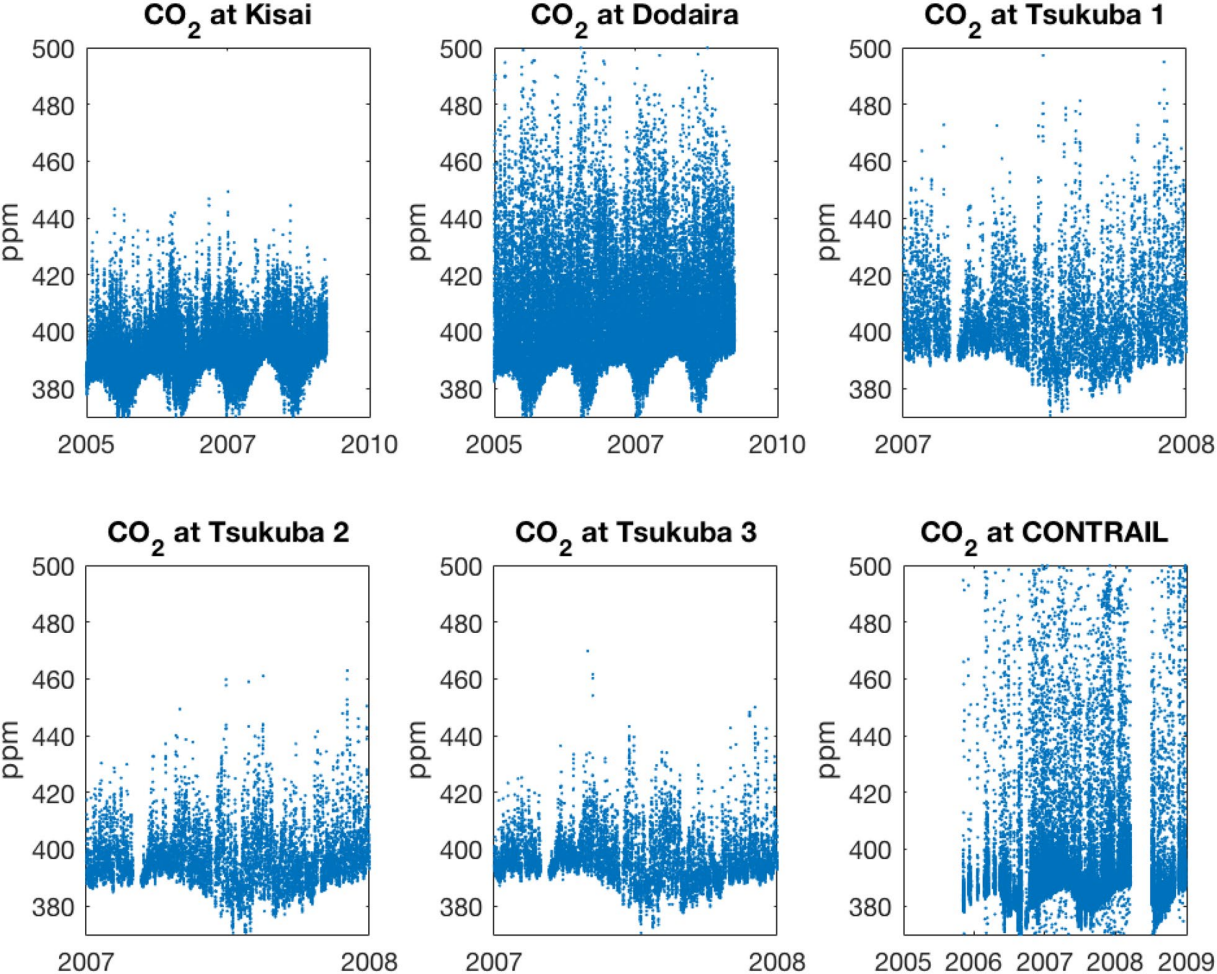
Values of the available CO_2_ measurements used for this study. The time series correspond to the stations at Kisai (13 m.a.s.l.) and Mt. Dodaira (840 m.a.s.l.), the three levels of the Tsukuba tower (base at 33 m.a.s.l., inlets at 25 m, 100 m and 200 m above ground level) and the composite of the CONTRAIL data (variable heights from ~ 500 m.a.s.l to 2000 m.a.s.l)

We have selected a set of measurements taken during the winter months for the years 2005 to 2009 for analysis. Figure 8 shows the spatial distribution of data used within the region. We have combined CO_2_ data from ground stations, a tall tower and in situ aircraft measurements covering the Tokyo Bay Area (Fig. 9). The total number of data points used in this work is 176,414. In situ high-resolution measurements being used include:

(i) Tsukuba tall tower measured CO_2_ mixing ratio in sampled air from inlets located at 25, 100, and 200 m. Introduced by the diaphragm pump to a nondispersive infrared sensor (NDIR) in the experimental field building [41, 42]. The mixing ratio standard used for calibration of the instrument was MRI-87 scale, described by Inoue and Matsueda [41]. The difference of MRI-87 standard and the World Meteorological Organisation (WMO) mole-fraction is less than 0.2 ppm for the ambient CO_2_ level, although it depends on the mixing ratios [43]. Tsukuba tall tower data were used hourly averaged for the whole year 2007. The Tsukuba tower was demolished and is no longer available.
(ii) The Comprehensive Observation Network for TRace gases by AIrLiner (CONTRAIL) project [44] provided the aircraft CO_2_ measurements. The project started in 2005 with two Boeing 747-400 aircraft and three 777-200ER aircraft operated by Japan Airlines (JAL) between Japan and Europe, Asia, Australia, Hawaii and North America. Further 777-200ER and 777-300ER aircraft were subsequently added. Samples were collected with the Continuous CO_2_ Measuring Equipment (CME) on board five different Japan Airlines (JAL) passenger aircraft during regular flights. CO_2_ measurements in the area of the Narita airport are used in this study during the ascending and descending parts of the flights (10 s averages). The measurements are reported in NIES-95 standard scale. Data are spans from mid 2005 to 2009.
(iii) Atmospheric CO_2_ hourly mixing ratio data from Mt. Dodaira and Kisai were obtained hourly averaged from the World Data Center for Greenhouse Gases (WDCGG) hosted by the Japan Meteorological Agency, Tokyo (Available at http://gaw.kishou.go.jp). A VIA-510R non-dispersive infrared absorption (NDIR) system is used at both sites [45]. The absolute scales of these measurements are WMO mole fraction scale and are calibrated by JMA secondary gas (reference gas: 390, 410, 430, 450 and span gas 380) the accuracy is 0.1 ppmv and the calibration frequency 2 h. The sites of WDCGG provide a continuous record of data; we chose data from 2005 to 2009 for this analysis.

There are therefore six simultaneous time series of measurements: Dodaira, Kisai, the three levels of the Tsukuba tower and CONTRAIL. We developed a workflow in order to combine data from different origins into a format that can be flexibly ingested by the model. Additional file 2: Figure S2 shows the number of data as a function of height.

Data were used as provided by the data generator. In the case of the two ground stations and the Tsukuba tower, the data provided was hourly averaged. CONTRAIL data was provided averaged every 10 s (see Additional file 6). Averaging CONTRAIL data hourly would result in the receptors becoming a long transect. As the cruise speed of the aircraft is ~ 900 km h^−1^, at 10 s time resolution, a typical distance between data points along the flight path is about 2.5 km, which is large compared to the fixed positions of the ground stations and the tower. The spatiotemporal scales covered are equivalent for a wind magnitude at the ground stations of about 2.5 km h^−1^ (0.7 m s^−1^), which lies within the normal range.

### Atmospheric composition and transport modeling

#### Lagrangian trajectories and particle dispersion models

We used two global scale Lagrangian trajectory codes: FLEXPART version 8.1 [21] and TRACZILLA [20, 24]. TRACZILLA is a FLEXPART branch derived from version 5 of FLEXPART. It was originally developed for large scale applications focussed on the Lagrangian trajectories themselves rather than with the gridded output as the main FLEXPART version. The method of Ensembles of Lagrangian Backward Trajectories was developed using TRACZILLA. It was used here to investigate the impact of the background calculation in the inversion results. Regional scale modeling was performed using FLEXPART-WRF [22] driven by the regional model WRF-CO_2_ [46, 47], based on WRF (Weather Research and Forecasting, [48]). Different meteorological advection fields have been used in this study. TRACZILLA and FLEXPART 8.1 are driven by ERA Interim (EI) global ECMWF reanalysis [49] at 1° × 1° spatial resolution (T255L spectral truncation) and 3 hourly time resolution. The Eulerian mesoscale model WRF-CO_2_ that drives FLEXPART-WRF was configured with two nested domains. The outer domain covers East Asia with 27 km grid spacing. The map projection used for the model domain was Lambert Conformal with 165 × 132 grid cells. The inner domain has a spatial resolution of 10 km and is centered at (35 N, 133 E), which is near Tokyo (Additional file 5: Figure S5). The model has 30 vertical layers up to 100 hPa, and 11 layers are located within 2 km above the ground level. The time resolution of the WRF runs was 1 h.

The transport and mixing processes determine the impact of the emission fluxes (the sources) on the measured concentration values (the receptors). Lagrangian trajectories are calculated for the estimation of the SRR only for the period the influence of the fluxes to be estimated is significant. The trajectories were calculated in the current study for all available receptors. One backward trajectory ensemble of 100 trajectories was calculated starting on every receptor measurement location (see “Measurements: CO_2_ mixing ratios measured in commercial aircraft, tower and surface stations” section on measurements) with a time resolution matching that of the measurements (1 h for ground observations, 10 s for airborne observations). For the computation, the ensembles associated with each observation were organised in groups defined by the observation date (i.e. all observations-trajectory ensembles for a period of 24 h). For each of these observations-trajectory ensembles groups, a FLEXPART simulation was run. A FLEXPART simulation can contain an arbitrary number of ensembles of trajectories (“releases”) associated with spatiotemporal observations. The release times can be defined with a one second time resolution. The gridded and particle output was stored every hour for post processing for the period necessary for the SRR calculation. For the gridded output, the residence times are stored that are a result of sampling the trajectories at the internal time step of the model. The synchronisation time of FLEXPART is 900 s for the advection and 18 s for the turbulent mixing. In general no more than the previous 3 days (and usually much less, of the order of 1.5 days) are necessary for flux estimation in the Tokyo Bay Area, as longer backward calculation would come from areas beyond. The reference inversion was calculated with 36-h trajectories. The positions of the particles are stored in order to have available the end positions of the trajectories in order to estimate the background by means of ensembles of diffusive backwards trajectories (see below).

#### Definition and calculation of the source receptor relationship

For the period under consideration (2005 to 2009), ensembles of trajectories associated with the measurements were processed to estimate the source–receptor relationship for all measurement points. The results are based on a 20 km × 20 km grid. Every day there are at least 48 rows in the SRR matrix corresponding to the two ground sites (from WDCGG, the World Data Centre for Greenhouse Gases) hourly data (averaged by the provider). The system required regularisation for the matrix inversion. The matrix is solved on a daily basis, allowing a faster calculation than a full matrix for all the data in the time series. This sets out a simple parallelisation methodology, and is justified by noting that the matrices are close to diagonal. For each day of measurements the algorithm can provide an estimate of fluxes for the previous period for which the trajectories are calculated. In general, the shapes of the clouds of points used to construct these matrices with different meteorologies show a consistent picture of transport (see Additional File 4: Figure S4 a and b and text in Additional File 6 for further details). The distance between the two operators calculated as the L^1^ norm (the sum of the absolute values) of the difference is about 10–15%. Each model’s meteorologies are based on different assimilation systems (ECMWF and NCEP—the National Centers for Environmental Prediction). The agreement between different transport models sets the stage for subsequent analysis. However, even if results obtained using ERA Interim and WRF meteorologies are consistent with each other, this cannot ensure that other errors cannot occur as discussed above (“Sensitivity to transport model errors” section). The time dependent SRR can be adapted to the estimate of the main fluxes directly (i.e. retrieval for static fluxes) by adding the columns with the residence times for the same emission regions. In that way the SRR matrix can be multiplied by a fixed set of mean emissions without time dependence in order to obtain a representation of the mixing ratio values with the forward model (see “The forward model” section).

#### Initial and boundary conditions: background concentrations

We use four different background definitions, (1) from the data (2) from the Mauna Loa measurements time series, (3) from a global model, and (4) from a perturbation to (2) and (3). The reference background can be obtained directly from the data, by taking the daily minimum for each ground site or using the free troposphere observations of CONTRAIL. A simplified methodology for background estimation is based on interpolating the value in a clean air ocean station (e.g. Mauna Loa in the Pacific) in order to estimate the hemispheric CO_2_ background concentration. Although coarse, this approach contains important information about the meridional and seasonal baseline concentration. This has the advantage of being quickly and easily calculated for any measurement. In addition, we calculated the flux resulting from perturbations to the backgrounds of ± 2 ppb. We have used a general circulation model for CO_2_ together with ensembles of diffusive backward trajectories (EDBTs), a previously developed method for Lagrangian tracer reconstructions [20, 24, 25, 50]. The global CO_2_ 3D fields are provided by the time dependent output of ACTM [11]. ACTM is the Atmospheric Chemistry Transport Model for simulations of long-lived gases in the atmosphere is based on the CCSR/NIES/FRCGC (Center for Climate System Research/National Institute for Environmental Studies/Frontier Research Center for Global Change) atmospheric general circulation model (AGCM). For a given spatiotemporal observation, EDBTs assign as background mixing ratio an average of values interpolated from the Eulerian model 3D output. These mixing ratio values are interpolated at the endpoints of the ensemble trajectories associated with the spatiotemporal observation. In this case the background value for each measurement location and time was calculated as the average of the interpolated ACTM values at the end of each member of the ensemble of 100 backward trajectories converging to the measurement location and time. Each measurement in space and time can be assigned different background values depending on how far back in time the background is defined (air mass considered “old” or “aged”). It is interesting to establish a comparison of the same framework applied to atmospheric flows with longer mixing and transport time scales. Those yield longer “background” time scales, e.g. up to several months in the upper troposphere. When the flow is adequately represented, the measurements can be accurately reconstructed [24, 51]. We have assessed different options for evaluating the boundary conditions in order to estimate the bias they can introduce in the flux estimates in the results and discussion sections. For all background methodologies, perturbations were calculated in order to assess the sensitivity.

#### Emission fluxes from inventory data

**Fig. 10:**
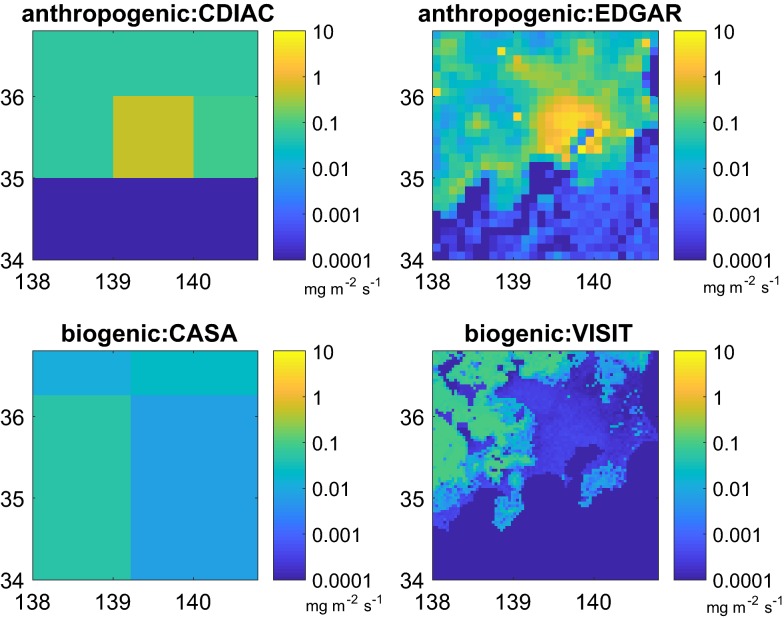
Inventories used in this study. Upper row (anthropogenic): CDIAC (left), EDGAR (right). Lower row (biogenic): CASA (left), VISIT (right). In winter, the anthropogenic emissions can reach 10 mg m^−2^ s^−1^ a much higher value than the biogenic fluxes that range below 0.1 mg m^−2^ s^−1^

Figure 10 shows the inventories used in this study. The a priori information for the anthropogenic fluxes is based on two different inventory data sets: from the Emissions Database for Global Atmospheric Research—EDGAR version 4.2 [4], and from the Carbon Dioxide Information Analysis Center—CDIAC [52]. EDGAR is developed by the Netherlands Environmental Assessment Agency and the European Commission’s Joint Research Centre. The database allows calculating emissions by country sector and includes specific technologies for combustion/processing and emission abatement measures. We used a resolution of 0.1° × 0.1° in this work. EDGAR is provided at yearly resolution. For the time series analysis, we used 2005 as a reference year. The CDIAC database is developed at The Oak Ridge National Laboratory (ORNL) and includes estimates of carbon dioxide emissions from fossil-fuel consumption and land-use changes; records of atmospheric mixing ratios of carbon dioxide and other trace gases that impact the radiative balance; carbon cycle and terrestrial carbon management datasets and analyses; global and regional climate data and time series; and analyses of land-cover/land-use change. CDIAC is provided by the U.S. Department of Energy (DOE). CDIAC is provided at yearly resolution and 1° × 1°. For the time series analysis, we used 2005 as a reference year.

The biogenic fluxes are based on two ecosystem models: CASA [53] and VISIT [27]. Carnegie-Ames-Stanford approach (CASA) is a terrestrial biosphere model that simulated monthly changes for carbon dioxide released into the atmosphere as microbes decompose plant debris in the Earth’s soil. The model simulates net primary production (NPP) and soil heterotrophic respiration (HR) at regional to global scales. Model outputs include the response of net CO_2_ exchange and other major trace gases in terrestrial ecosystems to inter-annual climate variability. CASA is available at monthly resolution at 1° × 1°. The Vegetation Integrative SImulator for Trace gases (VISIT) is an integrated model for simulating the biogeochemical interactions. It is designed as a component of Earth System Models, connected to them with physical interaction schemes. The model consists of carbon, nitrogen, and water cycling schemes, which consider mutual interactions and aims at simulating exchange of trace gases by terrestrial ecosystems. VISIT is provided at daily resolution at 1/30° × 1/30°. The inventory flux data was interpolated (or aggregated if higher resolution) into the model grid conserving the total mass emitted within the domain of interest (Fig. 10).

### Inverse modelling

#### The forward model

Atmospheric composition can be analysed as a combination of younger (e.g. recent surface emissions) and older (long range transport, background value) processes. The atmospheric CO_2_ mixing ratios at a set of given locations in space–time (that can be modelled or measured) can be represented as the vector $\varvec{CO}_{2}^{{\varvec{mix}}}$ as

1 $$ \varvec{CO}_{2}^{{\varvec{mix}}} = {\mathbf{SRR}} \varvec{CO}_{2}^{{\varvec{flux}}} + \varvec{CO}_{2}^{{\varvec{background}}} $$

where the vector $\varvec{CO}_{2}^{{\varvec{flux}}}$ contains the spatiotemporal surface emissions, the matrix **SRR** contains the average residence times in the grid cells where the fluxes occur of the air masses arriving at the locations where $\varvec{CO}_{2}^{{\varvec{mix}}}$ is sampled (observations and/or models) and the background $\varvec{CO}_{2}^{{\varvec{background}}}$ corresponds to the amount of CO_2_ present in air parcels before the fluxes take place. The SRR does not contain the values of the fluxes but only the sensitivity to their locations. Emission inventories provide information on CO_2_ fluxes (see description of priors in “Emission fluxes from inventory data” section) and are solved for in the context of an inverse model. Here, Lagrangian transport models are used to calculate the matrix ${\mathbf{SRR}}$ as described above.

#### The inversion algorithm

In this work we apply a Bayesian maximum a posteriori method based on a widely used formulation [54, 55]. In general, the Linear Least Squares criterion can be written as the minimisation of the cost function $J$ (e.g. Equation 3.32 of Tarantola [55] in a notation consistent with Ide et al. [56]).

$$ J\left( {\mathbf{x}} \right) = \left( {{\mathbf{Hx}} - {\mathbf{y}}^{{\mathbf{o}}} } \right)^{{\mathbf{T}}} {\mathbf{R}}^{ - 1 } \left( {{\mathbf{Hx}} - {\mathbf{y}}^{{\mathbf{o}}} } \right) + \left( {{\mathbf{x}} - {\mathbf{x}}^{{\mathbf{b}}} } \right)^{{\mathbf{T}}} {\mathbf{B}}_{0}^{ - 1} \left( {{\mathbf{x}} - {\mathbf{x}}^{{\mathbf{b}}} } \right) $$

where ${\mathbf{y}}^{{\mathbf{o}}} = \varvec{CO}_{2}^{{\varvec{measurements}}} - \varvec{CO}_{2}^{{\varvec{background}}}$ is the observation vector, the vector ${\mathbf{x}}^{{\mathbf{b}}}$ is the prior $\varvec{CO}_{2}^{{\varvec{flux}}}$ and the vector ${\mathbf{x}}$ is the $\varvec{CO}_{2}^{{\varvec{flux}}}$ being solved for (the 2D or 3D arrays are reshaped into 1D vectors). The linear operator **H** (the observation operator in Ide et al. [56]) corresponds to the source–receptor relationship **SRR**. The ${\mathbf{y}}^{{\mathbf{o}}}$ vector is for all sites at once. The SRR is calculated from Lagrangian trajectories covering the whole area). The vectors ${\mathbf{x}}$, ${\mathbf{x}}^{{\mathbf{b}}}$ contain the time dependency of the fluxes in case it is taken into account. Assuming that both prior and posterior probabilities are Gaussian, the centre and second moment of the posterior distribution are given by the following expressions from Tarantola [55], Eqs. 3.37 and 3.38, in a notation consistent with Ide et al. [56]:

$$ {\mathbf{x}} = {\mathbf{x}}^{{\mathbf{b}}} + {\mathbf{B}}_{0} {\mathbf{H}}^{{\mathbf{T}}} \left( {{\mathbf{HB}}_{0} {\mathbf{H}}^{{\mathbf{T}}} + {\mathbf{R}}} \right)^{ - 1} \left( {{\mathbf{y}}^{{\mathbf{o}}} - {\mathbf{Hx}}^{{\mathbf{b}}} } \right), $$

$$ {\mathbf{B}} = {\mathbf{B}}_{0} - {\mathbf{B}}_{0} {\mathbf{H}}^{{\mathbf{T}}} \left( {{\mathbf{HB}}_{0} {\mathbf{H}}^{{\mathbf{T}}} + {\mathbf{R}}} \right)^{ - 1} \left( {{\mathbf{HB}}_{0} } \right) $$

The different a priori inventory data are available for all measurements during the winter months (December, January, February and March) from 2005 to 2009. For every month, a local sub matrix with the rows corresponding to each day and the columns corresponding to the relevant emission regions was constructed. The algorithm is based on a pseudo inverse formulation. The calculations used MATLAB and the LAPACK set of linear algebra routines [57].

#### Observation errors covariance matrix: measurement and transport uncertainty

The uncertainty in the observations can be expressed in the measurement error covariance matrix **R**, composed of the sum of instrumental error plus representation error. In general, representation error is composed of the sum of the matrices for aggregation, advection and background/boundary values. Diagonal elements represent the error in each observation and off-diagonal elements representing the correlated errors between observations. The observations **y**^**o**^ used in the inversion are the differences between the observed mixing ratios and the contribution from advection of the background (or lateral boundary) mixing ratios as explained above. Following Thompson et al. [58] the measurement, transport and boundary errors are assumed to be correlated over space and time. When observations are not aggregated, aggregation errors are not taken into account. Therefore the observational error is

$$ {\text{Observations }}\left( {\text{R}} \right) \, = {\text{ Instrumental }}\left( {\text{E}} \right) \, + {\text{ Advection }}\left( {\text{F}} \right) $$

The diagonal of the instrumental error covariance matrix E is the instrumental variance of the averaged observations. For every individual ground site, the standard deviation of sub-hourly variations within an hourly time window was used as the observation error when available. This information was only available for the Tsukuba tower, and the typical average value found was between ~ 1 and 3 ppm. The same value was used therefore for the data form Kisai and Dodaira and for CONTRAIL. Different instruments are assumed to have uncorrelated errors. This is reflected in the correlation matrix as the nonzero entries are blocks around the diagonal. The degree of correlation between measurement errors is represented by an exponential function, exp(∆t/A) where ∆t is the difference in time between measurements and A is the temporal correlation scale length (0.5 days).

The advection error F is not included in the reference inversion, but can be represented based on uncertainties in surface residence reported by Brioude et al. [59]. For a typical run at mesoscale in complex terrain, 4 km horizontal resolution and 1 h time interval output, the average uncertainty and bias in surface residence time were found to be 24% and 11% respectively, using instantaneous wind as in the current case. Here, the model uses absolute concentrations and can be affected by transport errors (i.e. mixing height, convection, advection, diffusion, etc.). The transport error depends on the accuracy of the planetary boundary layer (PBL) height estimate, which varies throughout the day. The correlation between transport errors is represented by an exponential function, exp(− ∆t/A) where ∆t is the difference in time between measurements and A is the correlation time scale. We performed sensitivity tests for different representations of F, the default being the diagonal of F defined as (0.24 **y**)^2^. This is discussed in “Discussion” section including some elementary sensitivity calculations.

#### Prior flux error covariance matrix

Following Gerbig et al. [60] and Thompson et al. [58] the diagonal elements of B_0_ (also called S_prior_) are related to the squared errors for each of the state variables (fluxes in grid cells) and the off-diagonal elements are derived from the correlated errors between them. The correlation is described by an exponential function

$$ {\text{C}}\left( {{\text{d}},{\text{t}}} \right) = {\text{e}}^{{ - \Delta {\text{d}}/{\text{D }} - \Delta {\text{t}}/{\text{T }}}} $$

where ∆d is the distance between state variables and ∆t is the time interval between variables representing fluxes at the same location but at different points in time. The denominator D is the spatial correlation scale length. T is the temporal correlation scale interval. We used different errors and error correlations for land (urban and rural) and sea fluxes.

For the error variance, given the lack of error estimates for the EDGAR and CDIAC inventories we have tested a range of values for the prior error and the error covariance. The prior flux error (the “standard deviation”) is assumed to be 50% for land grid cells and 100% for sea grid cells. Anthropogenic sea carbon fluxes may be nonzero due to maritime traffic which is non negligible in the Tokyo bay. The values provided by Moriwaki and Kanda [28] are available for comparison in the urban areas and, although limited in spatial coverage, are consistent with the error estimate. For the water grid cells no flux measurements are available for comparison.

Following Lauvaux et al. [61] who estimated spatial and temporal correlations in the model-data mismatch for CO_2_ inversions, horizontal correlation lengths are of the order of 50 km based on the spatial scale of the minor semi-axis (approximately north–south) of the Tokyo megalopolis. Because of the relative lack of additional information, we chose a correlation length that is consistent with the size of the Tokyo urban area. An approximation for the order of magnitude of the “diameter” of the TBA is 100 km. This relatively large spatial scale favours the geographical structure of the prior in the posterior: a modelling choice that helps the interpretation of the posterior results. But it is challenging to provide an objective definition. There are strong vertical correlations in the boundary layer, particularly during the day. Temporal correlations are stronger than spatial correlations and can last for most of a day. Land correlation scales are assumed to be shorter, 50 km and 10 km for rural and urban areas respectively.

The denominator D (the spatial correlation scale) is 100 km for sea fluxes. We are not giving priority in this study to assess the anthropogenic ship emissions. The temporal correlation scale length T is 30 days for the sea emissions between 1 and 3 days for the rural emissions (consistent with the maximum length of the trajectories) and 12 h for the urban emissions. There is no diurnal cycle in the prior emissions. The temporal correlations do not apply to static fluxes retrievals. The correlation between different grid cell types (sea, rural and urban, see Additional file 1: Figure S1) is assumed to be zero for simplicity in the subsequent analysis. Additional file 7: Figure S6 shows the retrieved fluxes resulting from constructing the prior error covariance matrix with alternative diagonal and off-diagonal terms.

The magnitude of the error reduction can be defined as r = 1 − σ_posterior_/σ_prior_, where σ_posterior_ and σ_prior_ are the diagonal elements of the error covariance matrices B and B_0_ respectively [62]. By the usual definition of σ_posterior_ (from the covariance matrices **B**_**0**_ and **R** are positive definite and the SRR has positive entries) r is always between 0 and 1. It is maximal if σ_posterior_ = 0 and it is zero if σ_posterior_ = σ_prior_. Therefore r can be interpreted as a measure of the reduction in uncertainty in the posterior estimate of the flux after the introduction of the information contained in the measurements. The lower right panel in Fig. 1 shows the spatial distribution of r in a latitude/longitude map. The error reduction correlation coefficient with the areas where the prior flux is higher is 0.68 with a p value < 0.01.

## Additional files

**Additional file 1: Figure S1.** Detail of the masks used for space averaging and construction of the error covariance matrix. Upper row: rural, urban and sea domains. Lower row: areas corresponding to EDGAR grid cells with flux higher than 1 mg m^−2^ s^−1^ (left panel) and 0.1 mg m^−2^ s^−1^ (center panel), for reference. Right panel shows the inner domain used when averaging over all areas.

**Additional file 2: Figure S2.** Vertical distribution of the data used in this study. The peaks corresponding to Kisai (13 m.a.s.l.), Dodaira (840 m.a.s.l.), and three levels of the Tsukuba tower (base at 33 m.a.s.l., inlets at 25 m, 100 m, and 200 m above ground level) are apparent. The remaining data variably distributed in height correspond to CONTRAIL data. The lowest data near the polluted airport within the mixed layer and directly influenced by the runaway emissions where not included in the inversions. A higher layer of consistently high values around 1 km was also removed. Night data between 00:00 and 06:00 was only used in the sensitivity tests.

**Additional file 3: Figure S3.** Hourly distribution of the CONTRAIL data used in this study. Most flights depart or arrive during the day. Only hours between 06:00 and 24:00 are used for the standard inversion.

**Additional file 4: Figure S4.** S4a) Source receptor relationship matrix calculated with different winds. Left: panel ERA Interim. Center panel: WRF. Right panel: difference. Rows (vertical axis) represent measurements grouped by site and release time (D = Dodaira, K = Kisai, T1 = Tsukuba 25 m, T2 = Tsukuba 100 m, T3 = Tsukuba 200 m, C = CONTRAIL). Columns (horizontal axis) represent the Tokyo Bay Area spatiotemporal surface fluxes between 2007-01-11 00:00 and 2007-01-13 24:00 JST. This corresponds to trajectory ensembles released during 2007-01-13 and integrated 48 h backwards in time. Source regions are aggregated by prefecture in the Kanto area for this particular case totaling 9 regiones (7 prefectures, rest of the land and sea) to improve the visualization as higher resolution SRRs are usually sparser. The gaps (matrix entries with SRR = 0) correspond to source regions not reached by the backward trajectories, i.e. for which the measurements provide no constraint. Time resolution of the fluxes is 3 hours here, but can change between 1 hour and static. Color scale represents the source-receptor relationship value in hours (i.e., the residence time: a factor that depends on the footprint layer height gives the sensitivity in e.g. mg CO_2_ m^2^s^−1^)^−1^. The integrated difference is of the order of 15 % of the source-receptor relationship calculated either with WRF of with ERA Interim winds. S4b) Three hourly footprints corresponding to the SRR described above. First columns: ECMWF winds. Second column: WRF winds. Third column: Difference ECMWF minus WRF. The shift North East– South West is apparent. The wind situation depicted is not uncommon. Compare with Fig. 6, where the misalignment is artificially produced by shifting the SRR directly.

**Additional file 5: Figure S5.** Domains of the WRF model version used in this study. Left: outer domain, 25 km horizontal resolution. Right: inner domain, 10 km horizontal resolution.

**Additional file 6: Table S1.** Flux density in mgCO_2_ m^−2^ s^−2^. **Text S1.** Further details of the CONTRAIL observations. **Text S2.** Definition and calculation of the Source Receptor Relationship. **Text S3.** Sensitivity to other elements of the inversion system. **Table S2.** Including transport model errors in the observation covariance matrix: perturbations on the SRR matrix. **Table S3.** Including transport model errors in the observation covariance matrix: background representation. **Table S4.** Including transport model errors in the observation covariance matrix for 3 hourly resolved, averaged fluxes. **Text S4.** Emission inventories for the city of Tokyo. **Table S5.** Yearly estimates of emissions from the energy sector from the Tokyo Metropolitan Government. **Text S5.** Information coming from the retrieval and the emissions inventory.

**Additional file 7: Figure S6.** Impact of changing the off diagonal terms on the prior error covariance matrix. S6a) Reducing the correlations to 10 km for all grid cells: the error reduction still follows roughly the prior fluxes distribution due to the diagonal terms proportional to the fluxes. S6b) The off diagonal terms are zero and the diagonal terms constant and set by the maximum gridcell value (1-sigma = max over the domain). The uncertainty is reduced mainly around the location of the observations and the error reduction follows the flow of the Lagrangian trajectories driven by the meteorological winds.

## Abbreviations

ACTM: Atmospheric Chemistry Transport Model, the CCSR/NIES/FRCGC (Center for Climate System Research/National Institute for Environmental Studies/Frontier Research Center for Global Change) atmospheric general circulation model (AGCM)-based chemistry transport model has been developed for simulations of long-lived gases in the atmosphere
CASA: Carnegie-Ames-Stanford Approach
CDIAC: Carbon Dioxide Information Analysis Center
CONTRAIL: Comprehensive Observation Network for TRace gases by AIrLiner
ECMWF: European Center for Medium-Range Weather Forecasts
EDGAR: Emission Database for Global Atmospheric Research
FLEXPART: FLEXiblePARTicle model
GHG: greenhouse gas
JAMSTEC: Japanese Agency for Marine-Earth Science and Technology
NCEP: National Centers for Environmental Prediction
SRR: source–receptor relationship
STILT: Stochastic Time-Inverted Lagrangian Transport model
TBA: Tokyo Bay Area
TRACZILLA: a FLEXPART branch focused on trajectory modeling
VISIT: Vegetation Integrative SImulator for Trace gases
WDCGG: World Data Centre for Greenhouse Gases
WRF: Weather Research and Forecasting model
