# Supplementary figures and images for: Assessing Lagrangian inverse modelling of urban anthropogenic CO2 fluxes using in situ aircraft and ground-based measurements in the Tokyo area
Source: Carbon Balance Manag. 2019 May 17;14:6. doi: 10.1186/s13021-019-0118-8 (PMC7227294; doi:10.1186/s13021-019-0118-8)

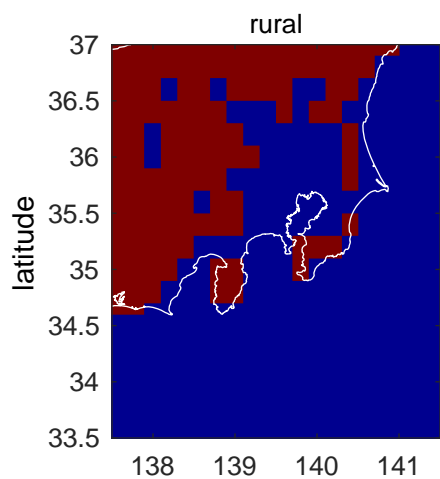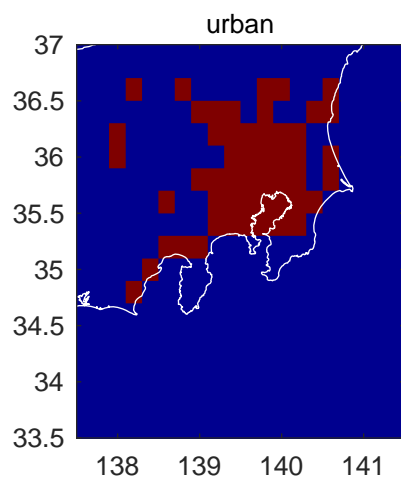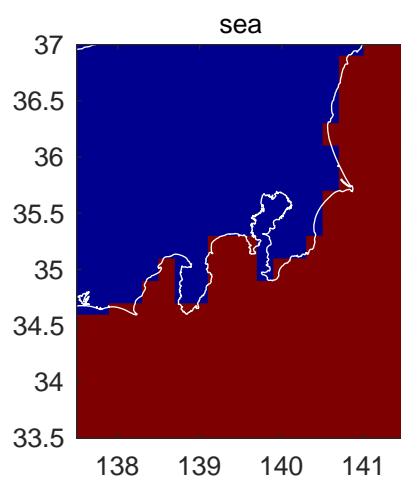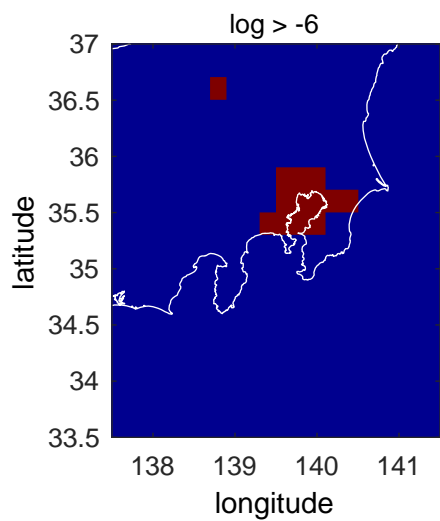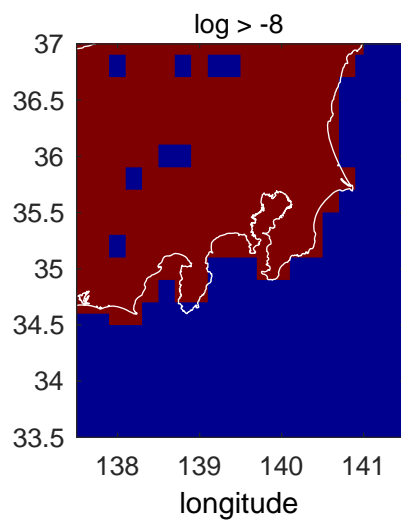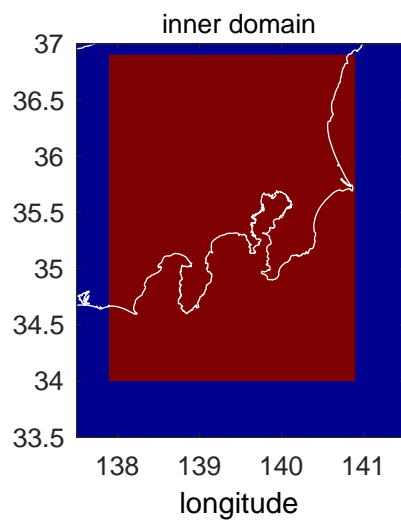

Supplement: Supplementary file 1 — Additional file 1: Figure S1. Detail of the masks used for space averaging and construction of the error covariance matrix. Upper row: rural, urban and sea domains. Lower row: areas corresponding to EDGAR grid cells with flux higher than 1 mg m−2 s−1 (left panel) and 0.1 mg m−2 s−1 (center panel), for reference. Right panel shows the inner domain used when averaging over all areas. [file 13021_2019_118_MOESM1_ESM.pdf]

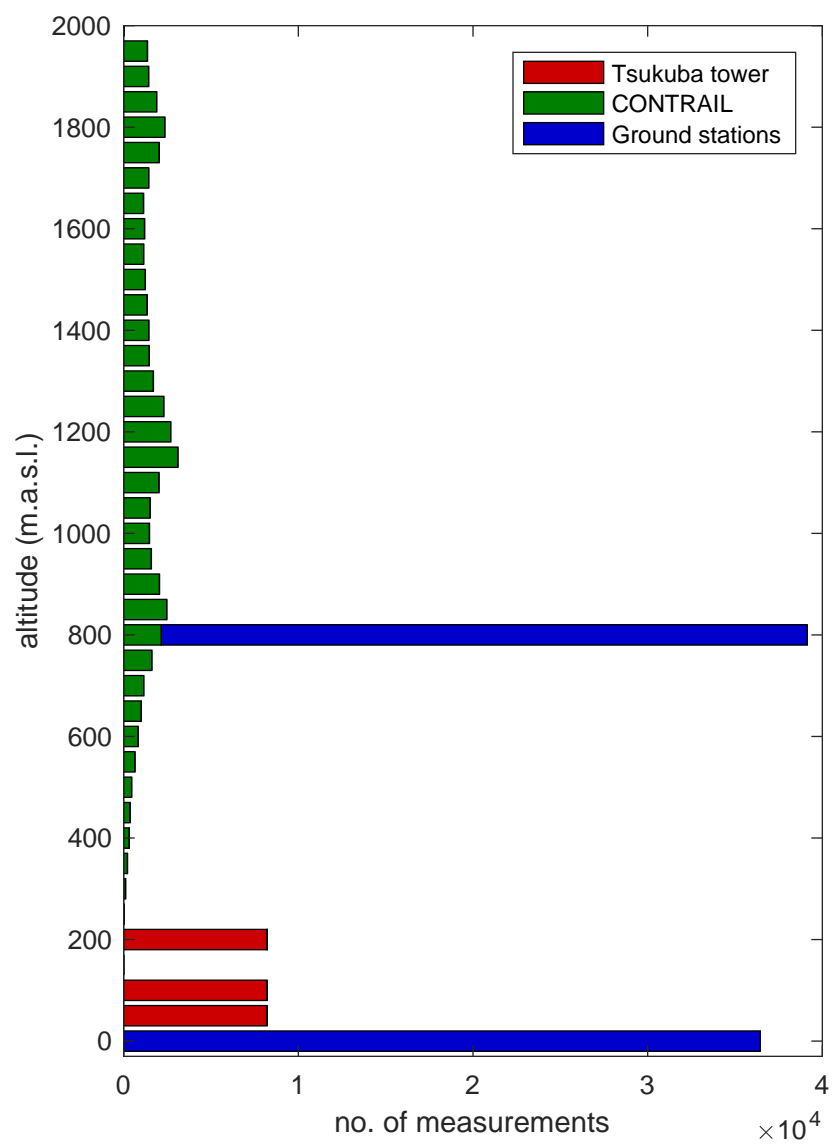

Supplement: Supplementary file 2 — Additional file 2: Figure S2. Vertical distribution of the data used in this study. The peaks corresponding to Kisai (13 m.a.s.l.), Dodaira (840 m.a.s.l.), and three levels of the Tsukuba tower (base at 33 m.a.s.l., inlets at 25 m, 100 m, and 200 m above ground level) are apparent. The remaining data variably distributed in height correspond to CONTRAIL data. The lowest data near the polluted airport within the mixed layer and directly influenced by the runaway emissions where not included in the inversions. A higher layer of consistently high values around 1 km was also removed. Night data between 00:00 and 06:00 was only used in the sensitivity tests. [file 13021_2019_118_MOESM2_ESM.pdf]

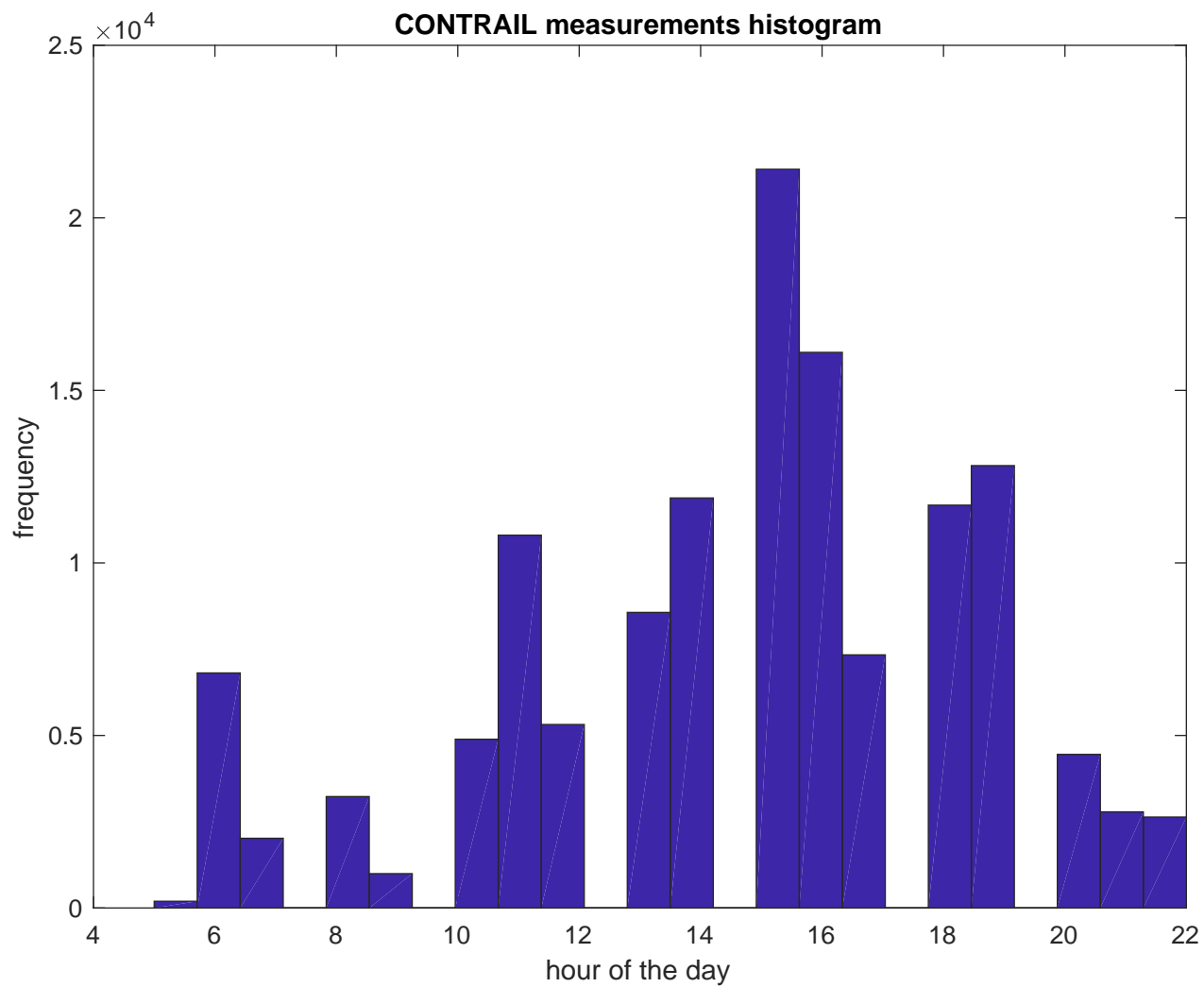

Supplement: Supplementary file 3 — Additional file 3: Figure S3. Hourly distribution of the CONTRAIL data used in this study. Most flights depart or arrive during the day. Only hours between 06:00 and 24:00 are used for the standard inversion. [file 13021_2019_118_MOESM3_ESM.pdf]

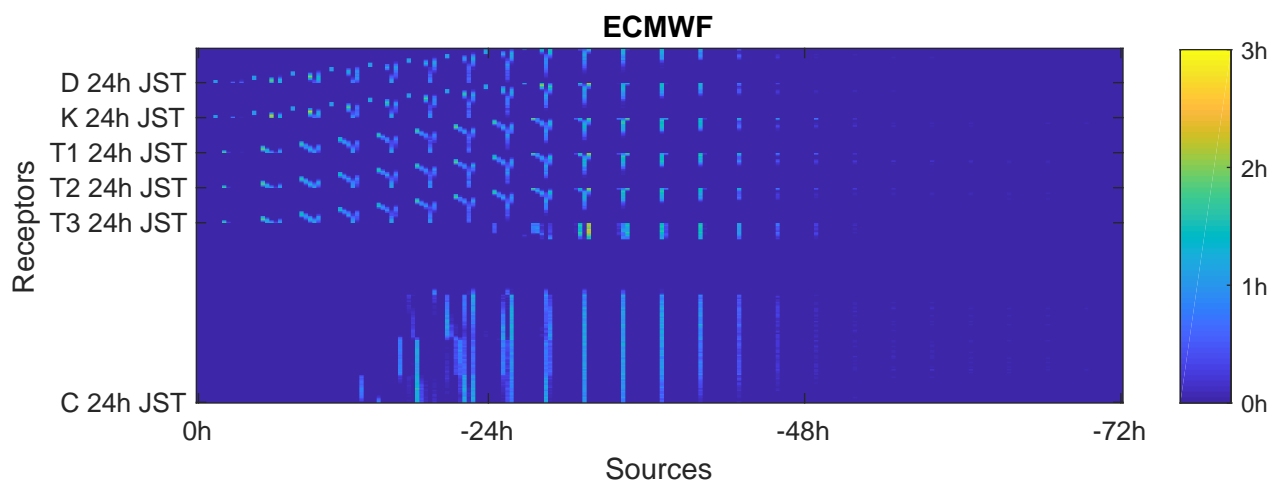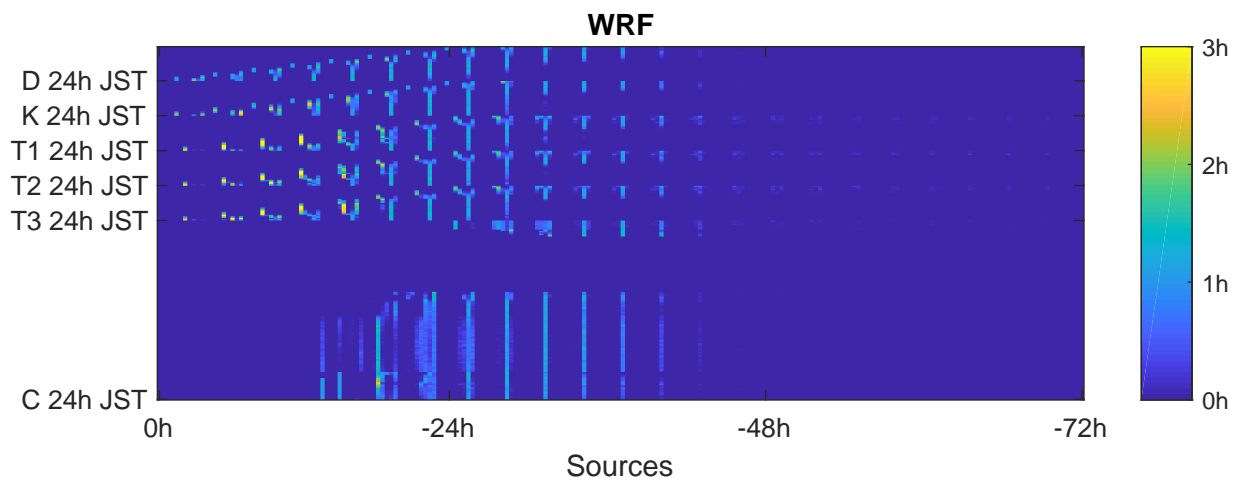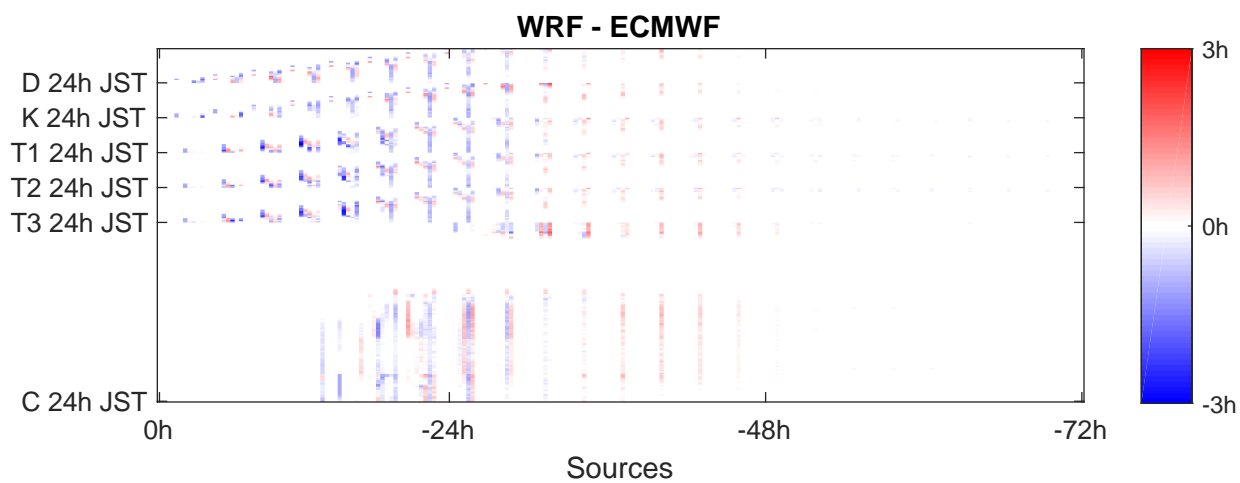

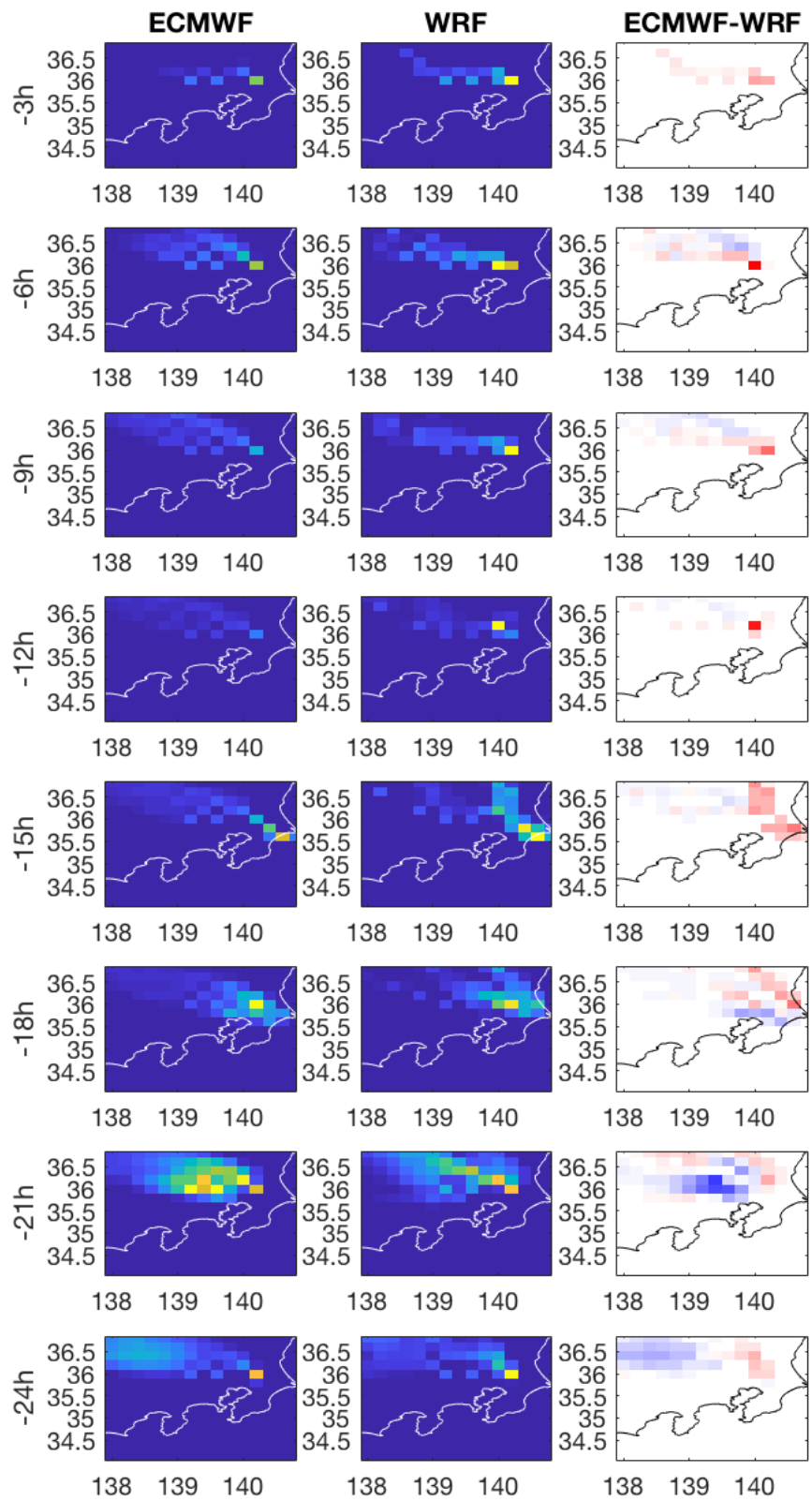

Supplement: Supplementary file 4 — Additional file 4: Figure S4. S4a) Source receptor relationship matrix calculated with different winds. Left: panel ERA Interim. Center panel: WRF. Right panel: difference. Rows (vertical axis) represent measurements grouped by site and release time (D = Dodaira, K = Kisai, T1 = Tsukuba 25 m, T2 = Tsukuba 100 m, T3 = Tsukuba 200 m, C = CONTRAIL). Columns (horizontal axis) represent the Tokyo Bay Area spatiotemporal surface fluxes between 2007-01-11 00:00 and 2007-01-13 24:00 JST. This corresponds to trajectory ensembles released during 2007-01-13 and integrated 48 h backwards in time. Source regions are aggregated by prefecture in the Kanto area for this particular case totaling 9 regiones (7 prefectures, rest of the land and sea) to improve the visualization as higher resolution SRRs are usually sparser. The gaps (matrix entries with SRR = 0) correspond to source regions not reached by the backward trajectories, i.e. for which the measurements provide no constraint. Time resolution of the fluxes is 3 hours here, but can change between 1 hour and static. Color scale represents the source-receptor relationship value in hours (i.e., the residence time: a factor that depends on the footprint layer height gives the sensitivity in e.g. mg CO2 m2s−1)−1. The integrated difference is of the order of 15 % of the source-receptor relationship calculated either with WRF of with ERA Interim winds. S4b) Three hourly footprints corresponding to the SRR described above. First columns: ECMWF winds. Second column: WRF winds. Third column: Difference ECMWF minus WRF. The shift North East– South West is apparent. The wind situation depicted is not uncommon. Compare with Fig. 6, where the misalignment is artificially produced by shifting the SRR directly. [file 13021_2019_118_MOESM4_ESM.pdf]

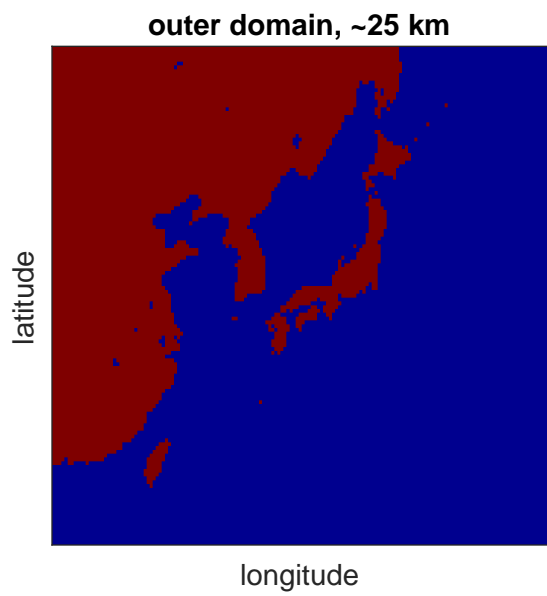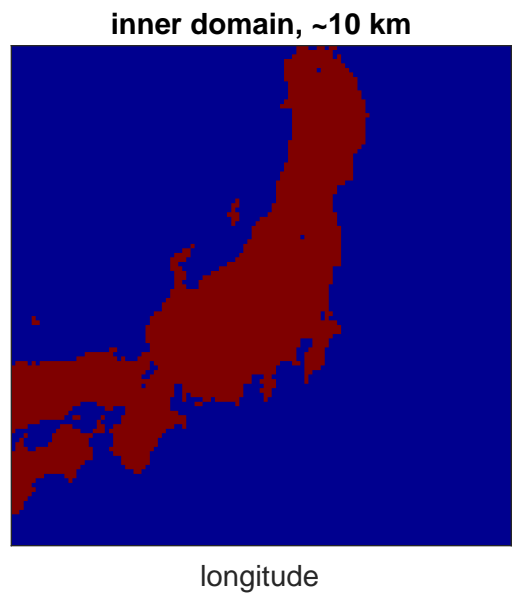

Supplement: Supplementary file 5 — Additional file 5: Figure S5. Domains of the WRF model version used in this study. Left: outer domain, 25 km horizontal resolution. Right: inner domain, 10 km horizontal resolution. [file 13021_2019_118_MOESM5_ESM.pdf]

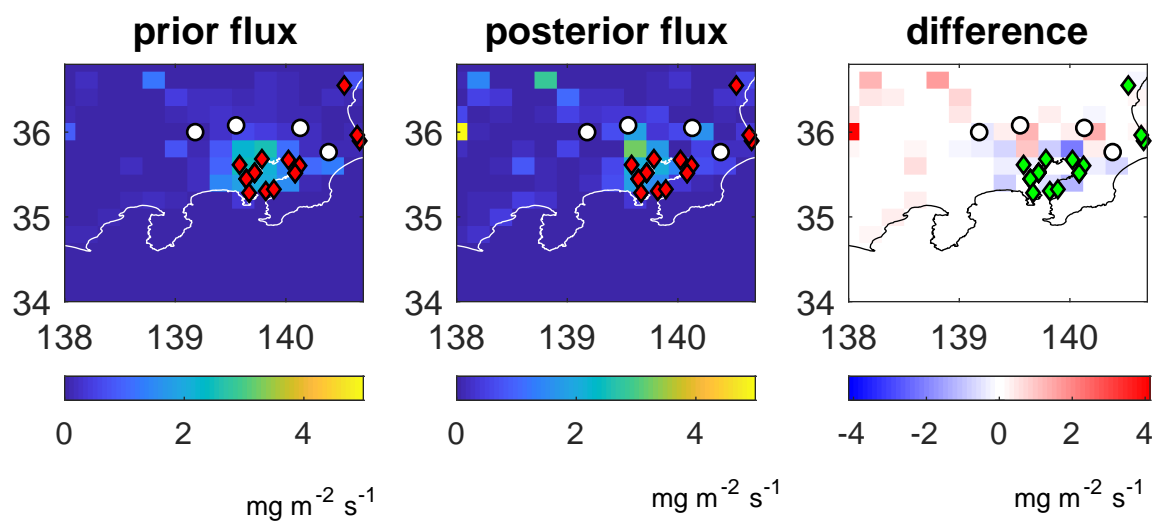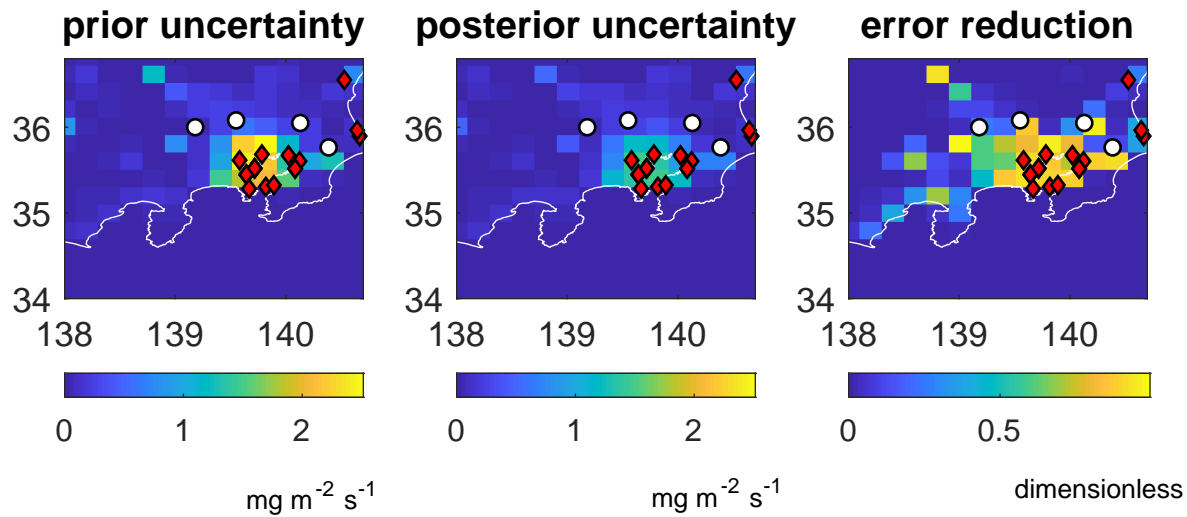

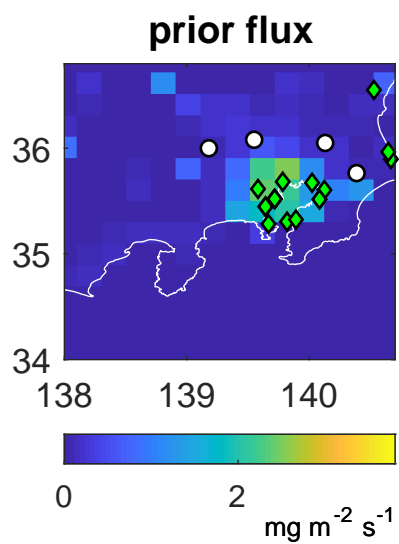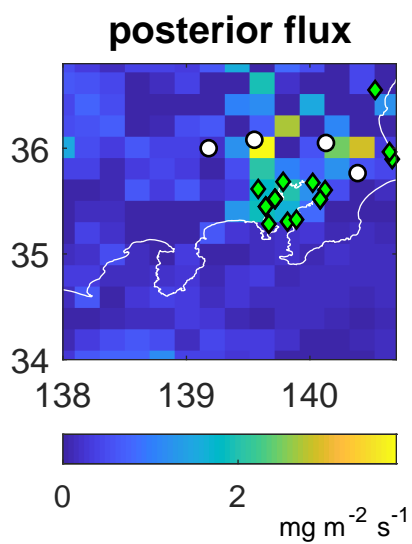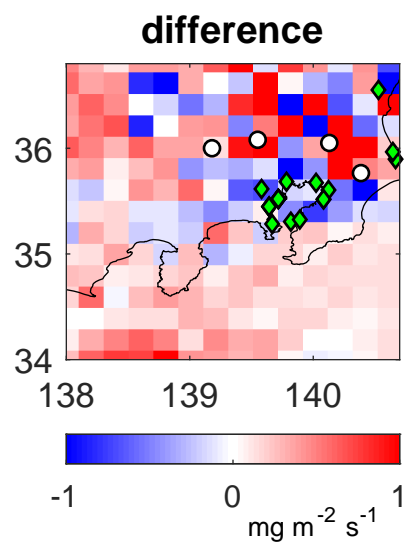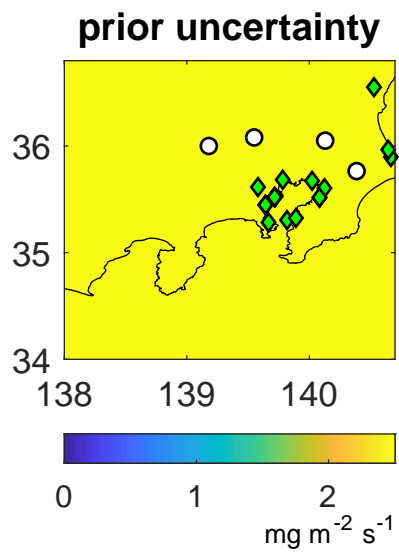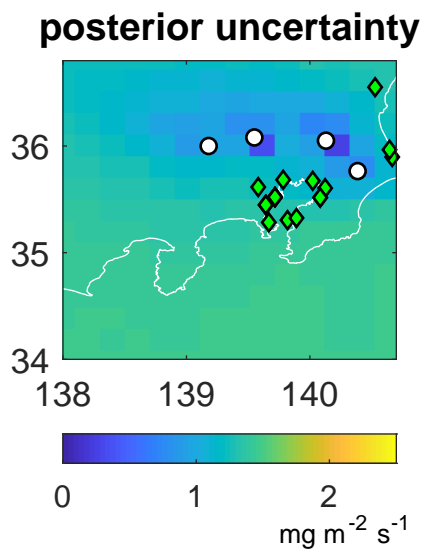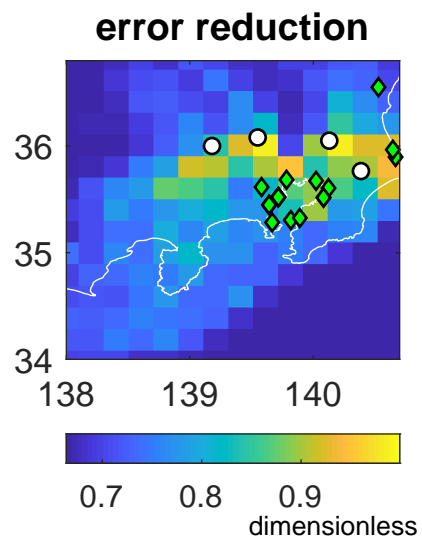

Supplement: Supplementary file 7 — Additional file 7: Figure S6. Impact of changing the off diagonal terms on the prior error covariance matrix. S6a) Reducing the correlations to 10 km for all grid cells: the error reduction still follows roughly the prior fluxes distribution due to the diagonal terms proportional to the fluxes. S6b) The off diagonal terms are zero and the diagonal terms constant and set by the maximum gridcell value (1-sigma = max over the domain). The uncertainty is reduced mainly around the location of the observations and the error reduction follows the flow of the Lagrangian trajectories driven by the meteorological winds. [file 13021_2019_118_MOESM7_ESM.pdf]
