# Supplementary material for: Assessing Lagrangian inverse modelling of urban anthropogenic CO2 fluxes using in situ aircraft and ground-based measurements in the Tokyo area
Source: Carbon Balance Manag. 2019 May 17;14:6. doi: 10.1186/s13021-019-0118-8 (PMC7227294; doi:10.1186/s13021-019-0118-8)
Supplement: Supplementary file 6 — Additional file 6: Table S1. Flux density in mgCO2 m−2 s−2. Text S1. Further details of the CONTRAIL observations. Text S2. Definition and calculation of the Source Receptor Relationship. Text S3. Sensitivity to other elements of the inversion system. Table S2. Including transport model errors in the observation covariance matrix: perturbations on the SRR matrix. Table S3. Including transport model errors in the observation covariance matrix: background representation. Table S4. Including transport model errors in the observation covariance matrix for 3 hourly resolved, averaged fluxes. Text S4. Emission inventories for the city of Tokyo. Table S5. Yearly estimates of emissions from the energy sector from the Tokyo Metropolitan Government. Text S5. Information coming from the retrieval and the emissions inventory. [file 13021_2019_118_MOESM6_ESM.docx]

Table S1. Flux density time and space averages for the whole period 2005 -2009. All values are in mg_CO2_ m^−2^ s^−1^ averaged in the area defined by the mask.

|  | prior flux | posterior flux | prior uncert | posterior uncert |
| --- | --- | --- | --- | --- |
| Rural | 0.04 | 0.05 | 0.02 | 0.02 |
| Urban | 0.64 | 0.86 | 0.32 | 0.25 |
| Sea | 0.01 | 0.01 | <0.01 | <0.01 |
| ≥ 1 mg m^-2^ s^-1^ | 1.71 | 1.80 | 0.85 | 0.65 |
| ≥ 0.01 mg m^-2^ s^-1^ | 0.27 | 0.37 | 0.13 | 0.11 |
| all grid | 0.17 | 0.23 | 0.08 | 0.07 |

### Text S1) Description of the CONTRAIL observations

**Measurement method**: Sample air is drawn by a diaphragm pump from an intake port connected to the air conditioning duct of the aircraft and introduced into an NDIR (LI-COR, LI-840) of the continuous measuring equipment (CME). Water vapor is removed from the sample air by a membrane dryer (Nafion Dryer) and magnesium perchlorate.

**Calculation method:** Sample air is continuously introduced to the NDIR except for time periods when the line is switched to standard gases. Two standard gases (CO_2_ in air), whose CO_2_ mole fractions are traceable to the NIES 09 scale, are introduced into the NDIR every 10-15 min during the ascent or descent portion of the flight and every 20-60 min during the cruise. The standard gases flow for 50 seconds and the last 10-second signals are averaged for the calculation. Sample air signals are averaged every 10 and 60 seconds for the ascent/descent and cruising portion of the flight, respectively. The CO_2_ mole fraction of sample air is calculated based on the signals of the standard gases taken before and after the sample signals. Sample measurements are corrected for the time lag of about 40 seconds between the intake of outside air and the measurement in the NDIR.

Figure S3 shows the histogram of arriving and departing times for the whole period used here.

### TextS2) Definition and calculation of the Source Receptor Relationship

The SRR only contains information about the atmospheric transport, namely the sensitivity to the (gridded) emissions (the emission inventories). It is by adding up the product between the SRR and the emission inventory that the atmospheric concentration enhancements are obtained. However the SRR is calculated for a particular spatiotemporal grid, so in that sense the geometry of the inventory is considered in the SRR. But the actual values of the emissions are never included in the SRR. Figure S4a represents an explicit example of a source-receptor relationship matrix. The rows of this matrix correspond to measurement points (in situ data) during a period of 24 hours. The columns represent emission sources (4 aggregated regions x 36 hours backwards.). Emission sources are aggregated gridded output of the Lagrangian model. In this case there are four aggregated regions for illustration purposes representing sea, urban, rural and rest of the land. Red color at an entry of the matrix means that this emission source (column) has a significant impact on the mixing ratio of the row (the measurement). Tick labels 'T1', 'T2', and 'T3' represent the different heights at the Tsukuba tower (at 25m, 100m and 200m above ground). These lines are the same lengths because the sites are hourly averaged and thus for a 24-hour period each has precisely 24 readings, which translates in 24 rows in the SRR (the same as the upper rows: for the ground sites of Kisai and Dodaira). For every site, the vertical axis can be interpreted as the t time dimension (forward) and the horizontal axis as the s time dimension (backwards) in the SRR. The lower rows correspond to the CONTRAIL data (averaged by the provider at 10 seconds). The blank rows in the middle correspond to measurements in the free troposphere that are not affected by local sources. Source-receptor relationship rows of zeros provide no constraint during the inversion process. This example of the source-receptor relationship matrix corresponds to the 1st of January 2007. Figure S4a also illustrates a decreasing influence of the emission regions as time evolves backwards as trajectories get diluted by transport and mixing and eventually the air masses move outside the domain. After 3 days the influence of the source regions is negligible and hence it is no longer taken into account. Mixing reduces the influence of far field sources, and therefore the Tokyo fluxes are dominant. Patterns calculated with ERA Interim and WRF winds show a fair agreement (Figure S4b).

### Text S3) Sensitivity to other elements of the inversion system

### Estimates of the prior error covariance

The constraint depends partly on the definition of the prior covariance matrix **B_0_**. If **B_0_** carries more weight than the observation error covariance matrix **R**, the observational constraint will be weaker. Figure S6 shows the impact of changing the off diagonal terms on the prior error covariance matrix. Figure S6a) Reducing the correlations to 10 km for all grid cells: the error reduction still follows roughly the prior fluxes distribution due to the diagonal terms proportional to the fluxes. Figure S6b) The off diagonal terms are zero and the diagonal terms constant and set by the maximum grid cell value (1-sigma = max over the domain). The uncertainty is reduced mainly around the location of the observations and the error reduction follows the flow of the Lagrangian trajectories driven by the meteorological winds.

### Time dependence of the fluxes

Another aspect of the transport definition is the time resolution of the SRRs. In addition to retrieval of daily means (with static fluxes) Different configurations of the SRR were tested between 1 and 24 hour resolution. In all cases, the total sensitivity expressed as residence time doesn’t change, but adding time intervals increases the dimensionality of the problem, reducing the ability of the observations to constrain the fluxes. The effect, in these cases in that the retrieved flux tends to be closer to the prior the higher the resolution used.

### Including transport model errors in the observation covariance matrix

The essential element of the error covariance is the instrumental errors represented by the matrix E described in section 5. These are generally of the order of 1 ppb for the observations used in this work. The simplest way of performing the optimizations is assuming no model errors. On the other hand, the transport errors can be included in the prior error covariance matrix of the observations **R**. However, increasing the observation error variances would weaken the constraint provided by the measurements. With the same prior flux covariance matrix, less weight is given to the observational constraint in favour of the prior fluxes. We explored the interplay between these effects by adding to **R** a matrix **F** containing in its diagonal the error estimates for the residence time in individual grid cells of 24% reported by Brioude et al. (2012). With the increased observation error covariance matrix, the impact of the shifted SRR in the retrieved fluxes is smaller than with **R** only containing the error of the measurements of 1 ppm for 1-sigma (Table S2).

Table S2. Including transport model errors in the observation covariance matrix: perturbations on the SRR matrix.

| **R with F (1-sigma=24%) transport** | | |
| --- | --- | --- |
| **Experiment Description** | **Kanto urban emissions Mt/month (all period average)** | **Relative difference with respect to reference** |
| Reference increased R | 37 | 0% |
| Shift right (increased R) | 36 | -1% |
| Shift left (increased R) | 36 | -1% |
| Shift up (increased R) | 36 | -1% |
| Shift down (increased R) | 37 | <1 % |

The transport uncertainty can be included also in the case of background perturbations in the observations error covariance as discussed above (see also methods section). Here we again added to the reference observation error covariance matrix **R** a matrix **F** containing 24% relative errors. With this modified setting we calculated the fluxes with the AGCM-EDBT and the ML derived backgrounds, and in addition these different backgrounds were perturbed by 2 ppm on either side (again considering 24% transport error). The results are shown in table S3. This illustrates how by reducing the dependency on the observations, the dependency on the prior increases and the dependence on the transport error decreases.

Table S3. Including transport model errors in the observation covariance matrix: background representation.

| **R with F (1-sigma=24%) background** | | |
| --- | --- | --- |
| **Experiment Description** | **Kanto urban emissions Mt/month (all period average)** | **Relative difference with respect to reference** |
| Reference increased R | 37 | 0% |
| AGCM (increased R) | 40 | 8% |
| AGCM-BDE -2 ppm (increased R) | 50 | 35% |
| AGCM-BDE +2 ppm (increased R) | 29 | -22% |
| ML (increased R) | 62 | 67% |
| ML -2 | 74 | 100% |
| ML +2 | 50 | 35% |

Finally, the reduced sensitivity to the observational constraint makes the posterior over reliant on the prior as seen in the sensitivity tests shown in Table S4.

Table S4. Including transport model errors in the observation covariance matrix for 3 hourly resolved, averaged fluxes. The posterior is much closer to the prior in this case, and the inversions don’t distinguish between very different priors.

| Fluxes and SRR resolution | | |
| --- | --- | --- |
| **Experiment Description** | **Kanto urban emissions Mt/month (all period average)** | **Relative difference with respect to reference** |
| Reference | 37 | 0% |
| CDIAC (3 hourly resolution) | 19 | -49% |
| EDGAR (3 hourly resolution) | 40 | 8% |
| 2x EDGAR (3 hourly resolution) | 70 | 89% |
| 3x EDGAR(3 hourly resolution) | 99 | 167% |

### Text S4) Emission inventories for the city of Tokyo

The bureau of environment of the Tokyo metropolitan Government publishes yearly reports of Energy Consumption and Greenhouse Gas Emissions in Tokyo every fiscal year (the current website is <http://www.kankyo.metro.tokyo.jp/en/climate/index.html>). The values can be found in section 3.2 (Total Greenhouse Gas Emissions) ,3.2.1 (Entire Tokyo), Table 3-5 Trends in total GHG emissions in Tokyo. The values correspond only the administrative region of Tokyo-tō (the capital district equivalent to a prefecture), and do not include surrounding areas that make part of the Tokyo megalopolis, like the cities of Kawasaki and Yokohama that belong the Kanagawa prefecture, south of Tokyo and the industrial docklands on the East side of the Tokyo bay that belong to the Chiba prefecture. Comparison of these values with the whole Kantō plain area requires additional hypothesis on the distribution and density of the fluxes.

Table S5. Yearly estimates of emissions from the energy sector from the Tokyo Metropolitan Government. Thses estimates correspond to the prefecture of Tokyo (Tokyo-tō) and do not include surrounding urbanised areas.

| Year | 2005 | 2006 | 2007 | 2008 | 2009 |
| --- | --- | --- | --- | --- | --- |
| MTCO2eq | 61.73 | 57.64 | 65.11 | 62.97 | 59.16 |

### Text S5) Information coming from the retrieval and the emissions inventory

We estimate how much information is coming from the retrieval and the emissions inventory in two ways:

1. by comparing the changes in the two terms of the cost function

$$J\left( \mathbf{x} \right)\mathbf{=}\left( \mathbf{Hx-}\mathbf{y}^{\mathbf{o}} \right)^{\mathbf{T}}\mathbf{R}^{\mathbf{-1}}\left( \mathbf{Hx-}\mathbf{y}^{\mathbf{o}} \right)\mathbf{+}\left( \mathbf{x-}\mathbf{x}^{\mathbf{b}} \right)^{\mathbf{T}}{\mathbf{B}_{\mathbf{0}}}^{\mathbf{-1}}\mathbf{(x-}\mathbf{x}^{\mathbf{b}}\mathbf{)}$$

before and after the retrieval and

1. by comparing directly the Euclidean norms of the observation-analysis mismatch $\left\| \mathbf{Hx}^{\boldsymbol{a}}\mathbf{-}\mathbf{y}^{\mathbf{o}} \right\|_{2}$and the analysis-background mismatch in the space of observations $\left\| \mathbf{Hx}^{\boldsymbol{a}}\mathbf{-}\mathbf{Hx}^{\boldsymbol{b}} \right\|_{2}$. The analysis $\mathbf{x}^{\boldsymbol{a}}$ is the same as the posterior and $\mathbf{x}^{\mathbf{b}}$ is the same as the prior.

For method 1 initially, with the prior as first guess ($\mathbf{x=}\mathbf{x}^{\mathbf{b}}$) the term in the flux space

$$J_{B_{0}}=\left\| \mathbf{x-}\mathbf{x}^{\mathbf{b}} \right\|_{\mathbf{B}_{\mathbf{0}}}=\left( \mathbf{x-}\mathbf{x}^{\mathbf{b}} \right)^{\mathbf{T}}{\mathbf{B}_{\mathbf{0}}}^{\mathbf{-1}}\mathbf{(x-}\mathbf{x}^{\mathbf{b}}\mathbf{)}$$

is zero, and the term in the observations space

$${J_{R}=\left\| \mathbf{Hx-}\mathbf{y}^{\mathbf{o}} \right\|}_{\mathbf{R}}=\left( \mathbf{Hx-}\mathbf{y}^{\mathbf{o}} \right)^{\mathbf{T}}\mathbf{R}^{\mathbf{-1}}\left( \mathbf{Hx-}\mathbf{y}^{\mathbf{o}} \right)$$

is $J_{R_{0}}=\left\| \mathbf{H}\mathbf{x}^{\mathbf{b}}\mathbf{-}\mathbf{y}^{\mathbf{o}} \right\|_{\mathbf{R}}$ (the 0 in R means initial).

After the retrieval, the two components become (f means final)

$$\left\| \mathbf{x}^{\boldsymbol{a}}\mathbf{-}\mathbf{x}^{\mathbf{b}} \right\|_{\mathbf{B}_{\mathbf{0}}}= J_{B_{f}}$$

the background-analysis mismatch B norm in the space of the fluxes and

$$\left\| \mathbf{Hx}^{\boldsymbol{a}}\mathbf{-}\mathbf{y}^{\mathbf{o}} \right\|_{\mathbf{R}}= J_{R_{f}}$$

the observation analysis mismatch norm R in the space of observations.

We estimate the proportion of the information coming from the observation as

$$I_{obs}= \frac{J_{R_{0}}-J_{R_{f}}}{J_{R_{0}}-J_{R_{f}}+J_{B_{f}}}$$

and the proportion of the information coming from the prior as

$$I_{prior}= \frac{J_{B_{f}}}{J_{R_{0}}-J_{R_{f}}+J_{B_{f}}}$$

We obtained a mean of $I_{obs}$of 0.53 and for $I_{prior}$ of 0.47 for the reference inversion in January 2007.

On the other hand, on for the same period the average of the Euclidean (method 2) norm of the observation-analysis mismatch is ~ 106 versus ~84 for the analysis-background mismatch (proportions of 45% vs. 55%). Not smaller, but of the same order of magnitude. Therefore, we conclude that the amount of information from the prior and the observational constraint is balanced in this case. Other months show similar numbers.
